# Diurnal variation in mesophyll conductance and its influence on modelled water-use efficiency in a mature boreal *Pinus sylvestris* stand

**DOI:** 10.1007/s11120-019-00645-6

**Published:** 2019-05-23

**Authors:** Zsofia R. Stangl, Lasse Tarvainen, Göran Wallin, Nerea Ubierna, Mats Räntfors, John D. Marshall

**Affiliations:** 1 Department of Forest Ecology and Management, Swedish University of Agricultural Sciences, Umeå, Sweden; 2 Department of Biological and Environmental Sciences, University of Gothenburg, Gothenburg, Sweden; 3 Research School of Biology, The Australian National University, Canberra, ACT Australia

**Keywords:** Carbon isotope ratio, Cavity ring-down absorption spectrometry, Online discrimination, Vapour pressure deficit, Photosynthesis

## Abstract

Mesophyll conductance (*g*_m_) is a critical variable for the use of stable carbon isotopes to infer photosynthetic water-use efficiency (WUE). Although *g*_m_ is similar in magnitude to stomatal conductance (*g*_s_), it has been measured less often, especially under field conditions and at high temporal resolution. We mounted an isotopic CO_2_ analyser on a field photosynthetic gas exchange system to make continuous online measurements of gas exchange and photosynthetic ^13^C discrimination (Δ^13^C) on mature *Pinus sylvestris* trees. This allowed the calculation of *g*_m_, *g*_s_, net photosynthesis (*A*_net_), and WUE. These measurements highlighted the asynchronous diurnal behaviour of *g*_m_ and *g*_s_. While *g*_s_ declined from around 10:00, *A*_net_ declined first after 12:00, and *g*_m_ remained near its maximum until 16:00. We suggest that high *g*_m_ played a role in supporting an extended *A*_net_ peak despite stomatal closure. Comparing three models to estimate WUE from ∆^13^C, we found that a simple model, assuming constant net fractionation during carboxylation (27‰), predicted WUE well, but only for about 75% of the day. A more comprehensive model, accounting explicitly for *g*_m_ and the effects of daytime respiration and photorespiration, gave reliable estimates of WUE, even in the early morning hours when WUE was more variable. Considering constant, finite *g*_m_ or *g*_m_/*g*_s_ yielded similar WUE estimates on the diurnal scale, while assuming infinite *g*_m_ led to overestimation of WUE. These results highlight the potential of high-resolution *g*_m_ measurements to improve modelling of *A*_net_ and WUE and demonstrate that such *g*_m_ data can be acquired, even under field conditions.

**Electronic supplementary material:**

The online version of this article (10.1007/s11120-019-00645-6) contains supplementary material, which is available to authorized users.

## Introduction

Mesophyll conductance (*g*_m_) describes the ability of CO_2_ to diffuse across the interior of the leaf. In plants with C_3_ photosynthesis, *g*_m_ is roughly similar in magnitude to stomatal conductance (*g*_s_), frequently accounting for about 40% of the decline in CO_2_ concentration from the ambient atmosphere to the chloroplasts (*C*_c_) (Flexas et al. 2008; Warren 2008a). As a consequence, it has an important place in leaf-level photosynthesis models (von Caemmerer 2000; Dewar et al. 2017), but has been so infrequently quantified that it is seldom included in earth-system models (Rogers et al. 2017). It also has a critical role in the inference of water-use efficiency (WUE) from stable carbon isotope composition (δ^13^C) of plant tissues or, conversely, in the inference of δ^13^C from gas exchange (Rogers et al. 2017). This role is caused by the decrease in CO_2_ concentration at the enzyme rubisco, where δ^13^C is determined, relative to the substomatal cavities, where WUE is determined. Mesophyll conductance provides a means to calculate this difference. If *g*_m_ could be accounted for, then δ^13^C could provide independent tests of the WUE predictions of leaf (von Caemmerer 2000; Wei et al. 2014), canopy (Keenan et al. 2013), and earth-system models (Rogers et al. 2017).

One reason for the relative paucity of *g*_m_ data is that it is more difficult to estimate than *g*_s_. Stomatal conductance to CO_2_ is easily estimated from humidity, temperature and transpiration measurements, which might come from leaf-level gas exchange or sap-flux data. Given the relative ease of making such measurements, high temporal resolution *g*_s_ data are available for many species and sites, and models of *g*_s_ based on theory and empirical data have converged (Medlyn et al. 2011) and been incorporated into global models (Prentice et al. 2014; Rogers et al. 2017). In contrast, measuring *g*_m_ requires simultaneous measurements of gas exchange and either chlorophyll fluorescence or photosynthetic discrimination against ^13^C (∆^13^C). Discrimination can be inferred from the δ^13^C signature of photosynthesis products, e.g. leaf soluble sugars, phloem contents, plant tissues (e.g.: Hu et al. 2010; Ubierna and Marshall 2011), or directly from leaf CO_2_ flux (e.g.: Evans et al. 1986; Warren et al. 2003; Bickford et al. 2009; Wingate et al. 2007, 2010; Maseyk et al. 2011; Campany et al. 2016). These methods are technically challenging, especially under field conditions, so that measurements are often made with low temporal resolution.

It has been difficult to model *g*_m_, because previous studies have found that *g*_m_ and *g*_s_ respond differently to changes in environmental conditions, suggesting that the two are not tightly coupled. Rapid responses of *g*_m_ have been described to several environmental variables (for a review see Flexas et al. 2008; Warren 2008a; Flexas et al. 2012). These variables include light intensity or quality (Flexas et al. 2007; Tholen et al. 2008; Hassiotou et al. 2009; Loreto et al. 2009; Campany et al. 2016), intercellular CO_2_ concentration (*C*_i_) (Flexas et al. 2007; Hassiotou et al. 2009; Vrábl et al. 2009; Bunce 2010; Douthe et al. 2011; Tazoe et al. 2011), and leaf temperature (Bernacchi et al. 2002; Yamori et al. 2006; Warren 2008b; Evans and von Caemmerer 2013). If *g*_s_ responded to other variables, or at different rates, then the ratio *g*_m_/*g*_s_ would change. For example, it has been shown that *g*_m_ responds similarly, but more quickly, to variable *C*_i_ than *g*_s_ (Flexas et al. 2007). In addition, the *g*_m_*/g*_s_ ratio was found to be temperature dependent in a study exploring the thermal acclimation of *g*_m_ in spinach (Yamori et al. 2006).

Vapour pressure deficit (VPD) is particularly interesting in this context, because *g*_s_ responds so strongly to it (Marshall and Waring 1984; Oren et al. 1999; Medlyn et al. 2011). In contrast, the response of *g*_m_ to VPD has not been extensively studied and the results so far are contradictory (Bongi and Loreto 1989; Warren 2008c, Loucos et al. 2017). Both temperature and VPD change dynamically under natural conditions, diurnally and seasonally, potentially influencing the *g*_s_ to *g*_m_ relationship. However, the magnitude and importance of this variability has yet to be explored.

Given constant *g*_s_, an increase in *g*_m_ would increase water-use efficiency (WUE) (Flexas et al. 2010; Galmés et al. 2011), which is defined as the ratio of net carbon assimilation (*A*_net_) to water loss through transpiration (*E*). This would happen because an increase in *g*_m_ has no direct effect on transpiration, but it increases photosynthesis, resulting in an increase of the *A*_net_/*E* ratio. Accounting for *g*_m_ is especially important when estimating WUE from δ^13^C. For example, WUE is often inferred from historic tree-ring isotope data (Marshall and Monserud 1996; Seibt et al. 2008; Voelker et al. 2016). Such inferences require that some value for *g*_m_ be assumed. This assumption is often embedded as a constant, empirical adjustment in the relationship between *C*_i_*/C*_a_ and isotopic discrimination (Farquhar et al. 1982), or extrapolated based on its correlation with *g*_s_ in models of WUE (Klein et al. 2015), although as noted above, the correlation with g_s_ is not always strong.

In this manuscript, we present continuous, simultaneous measurements of shoot-scale gas exchange and ^13^C discrimination in a 100-year-old *Pinus sylvestris* stand in northern Sweden. We use these simultaneous data streams to obtain hourly *g*_m_ estimates parallel to *g*_s_*, A*_net_ and *E*. We begin with a brief description of how the data were treated and evaluate the accuracy of our measurement system. We next explore the diurnal dynamics of *g*_s_ and *g*_m_ and their relationship to *A*_net_. Finally, we compare estimates of WUE derived from gas exchange (WUE_G_) with estimates derived from photosynthetic discrimination (WUE_∆_). Three photosynthetic discrimination (∆^13^C) models were used to calculate WUE_∆_: a comprehensive model, a partial model and a simple model. Additionally, the comprehensive model was applied using three different assumptions for *g*_m_ values. We compare the different models and calculations and discuss their impact on WUE_∆_ estimates.

## Materials and methods

### Description of the experimental site

The study was conducted in a ~ 100-year-old, naturally regenerated, even-aged stand of *Pinus sylvestris* (Scots pine) at the Rosinedalsheden experimental forest in northern Sweden (64°10′N, 19°45′E, 153 m above see level), during the growing season of 2017. The Rosinedalsheden experiment includes an intensive fertilisation treatment (Lim et al. 2015), but the current study was conducted entirely on the unfertilised area. The photosynthetic season typically extends from mid-April to mid-November, buds burst at the end of May, and stem diameter-growth ceases in late August (Tarvainen et al. 2018). The June to August mean temperature was 12.4 ± 0.8 °C (mean ± SD) and the mean monthly precipitation was 67.9 ± 8.6 mm (mean ± SD), based on the 15-year (2003–2017) data measured at the Vindeln-Sunnansjönäs meteorological station (Swedish Meteorological and Hydrological Institute, www.smhi.se) approximately 5 km from the experimental site. The site has weakly podzolised fine sandy soil with a thin (2–5 cm) organic layer (Hasselquist et al. 2012). The leaf area index was 2.7 and the average tree height was 18.6 ± 2.3 m (mean ± SD) in 2013 (Lim et al. 2015).

### Experimental setup for continuous measurements of gas exchange

Shoot gas exchange (CO_2_ and H_2_O) was measured continuously on one 1-year-old upper canopy shoot on four trees. A 16-m tall scaffolding tower was used to reach the shoots and secure the equipment. The shoot-scale gas exchange was measured using a custom-built multichannel gas exchange system (GUS) (Wallin et al. 2001; Tarvainen et al. 2016), equipped with infrared gas analysers (IRGA, CIRAS-1, PP systems Hitchin Herts, U.K.) to measure CO_2_ and H_2_O partial pressure in the air from shoot cuvettes and reference channels. The 330 ml shoot cuvettes had a transparent acrylic plastic (Plexiglas) top for natural illumination. The cuvettes were temperature (T) controlled to track the ambient T and were equipped with a light sensor (PAR-1 M, PP systems, Hitchin, Herts, UK). The polyethylene tubing that connected the cuvettes to the IRGAs were insulated and heated with cables to avoid condensation. Nonetheless, morning condensation could occur in the cuvettes in connection with heavy rain events; we filtered those days out in the current analysis. The GUS cycled through the four shoot cuvettes and two non-cuvette lines once per hour, spending 7 min at each position, which were divided into 2 min of waiting time to allow instrument readings to stabilise and 5 min of measurement. We used the means from the 5-min measurement periods in the subsequent analyses, which yielded approximately one value/cuvette/hour throughout the 9 days. The non-cuvette lines were used for data quality assurance and for measurement of δ^13^C of ambient air (see details in next chapter). The IRGAs were calibrated with 400 µmol mol^−1^ CO_2_ gas at the beginning and at the end of the growing season. Additionally, every hour the IRGAs were zero calibrated and the system ran a cross-calibration protocol to match values in the sample and reference channels.

### Continuous measurement of δ^13^C

The isotopic composition of the CO_2_ in the air entering and leaving the cuvettes was analysed with a cavity ring-down spectrophotometer (CRDS; G2131-i, Picarro Inc., California, USA). The CRDS was connected to the same central line as the GUS, in parallel to the sample IRGA. We tested the instrument at varying CO_2_ and H_2_O vapour concentrations and found that the δ^13^C values were dependent on both, with an asymptotic relationship of δ^13^C to CO_2_ concentration (Fig. S1) and a linear dependency to H_2_O vapour concentration (Fig. S2). The continuous δ^13^C readings were corrected to account for the CO_2_ and H_2_O concentration effects before the data were used in further analyses. The CRDS was factory-calibrated in 2017 and manually calibrated once per week, using two reference gases with known CO_2_ concentrations (411 µmol mol^−1^, SD = 5.1; 1606 µmol mol^−1^, SD = 13.1) and δ^13^C values (− 32.36‰, SD = 0.09; − 4.14‰, SD = 0.06). The reference gases were analysed at the SLU Stable Isotope Laboratory (Umeå, Sweden) with GB-IRMS (Gasbench II—Isotope Ratio Mass Spectrometer, Thermo Fisher Scientific, Bremen, Germany), which was calibrated against IAEA-co-9 and NBS 19 standards. We found the weekly calibrations to be sufficient, because the reference δ^13^C values were stable over the season (Fig. S3) and were offset from the reference gases by a constant 4.17‰ (SD = 0.1), after correction for CO_2_ concentration. The CRDS recorded δ^13^C values once per second during the 5-min calibration period, which were then combined into a mean for each calibration date and these means yielded standard deviations of 0.1‰ for δ^13^C.

### Calculation of leaf gas exchange parameters and mesophyll conductance

In this paper, we present data collected on nine sunny days during the summer (28th of June—2nd of July and 6th of July—9th of July), with daily minimum and maximum temperatures of 6.2 ± 0.5 °C and 24.4 ± 0.6 °C, respectively, and daily maximum irradiation of 1964 ± 25 µmol m^−2^ s^−1^. Because of the high latitude and season, sunrise was typically around 02:15 and sunset was around 23:00. These days were chosen for high photosynthetic rates and lack of condensation in the cuvettes and tubing. We optimised the system setup to yield clear and consistent δ^13^C values with the CRDS, using 5-min integrations at approximately 1-second intervals. Because each of these measurements contributed to the mean δ^13^C value, it was appropriate to calculate the standard error of the mean from them. This yielded high precision, typically SE < 0.06‰.

*A*_net_, *E*, *g*_s_, and *C*_i_ were calculated from the gas exchange data according to the model described by Farquhar et al. (1980). Boundary layer conductance has previously been found to be high (8.1 mol H_2_O m^−2^ s^−1^) (Uddling and Wallin 2012) in our gas exchange cuvettes, therefore, we assumed boundary layer resistance to be insignificant. Needles from the shoots enclosed in the cuvettes were collected at the end of the study campaign to determine the projected leaf area using a flat bed scanner (Epson 1600) equipped for dual scanning, and WinSEEDLE Pro 5.1a (Regent Instruments, Canada) analysis software.

Mesophyll conductance (*g*_m_) and *C*_c_ were estimated from the carbon isotope discrimination data collected by the CRDS. The *g*_m_ was calculated from the comprehensive ∆^13^C model of Farquhar and Cernusak (2012) that includes ternary corrections. In particular, we used the formulation of Evans and von Caemmerer (2013) (see supplementary materials for details) that calculates *g*_m_ as

1 $$ g_{\text{m}} = \frac{{ {\frac{1 + t}{1 - t}\left( {b - a_{\text{m}} - \frac{{eR_{\text{d}} }}{{A_{\text{net}} + R_{\text{d}} }}} \right)\frac{{A_{\text{net}} }}{{C_{\text{out}} }}} }}{{\Delta _{\text{i}} -\Delta _{\text{o}} -\Delta _{\text{e}} -\Delta _{\text{f}} }} $$

where *b, a*_m_ and *e* are the fractionation factors during carboxylation (*b* = 29‰), dissolution and diffusion through water (*a*_m_ = 1.8‰) and respiration (*e*, see Eqn. S5), respectively. *R*_d_ is daytime respiration (Eqn. S1), and *C*_out_^1^ is the CO_2_ concentration in the cuvette; ∆_i_, ∆_o_, ∆_e_ and ∆_f_ are, respectively, the discrimination when *C*_i_ = *C*_*c*_ (Eqn. S2), the observed discrimination during gas exchange (Eqn. S3 and S4), the discrimination associated with respiration (Eqn. S5 and S6) and with photorespiration (Eqn. S7). The term *t* is the ternary correction factor (Eqn. S8, Farquhar and Cernusak 2012). Note that *C*_out_ is lower than the atmospheric CO_2_ concentration (*C*_in_), due to *A*_net_ within the cuvette. The CO_2_ concentration at the site of carboxylation (*C*_c_) was calculated from *g*_m_ through the following relationship:

2 $$ A_{\text{net}} = g_{\text{m}} \left( {C_{\text{i}} - C_{\text{c}} } \right) $$

**Fig. 1:**
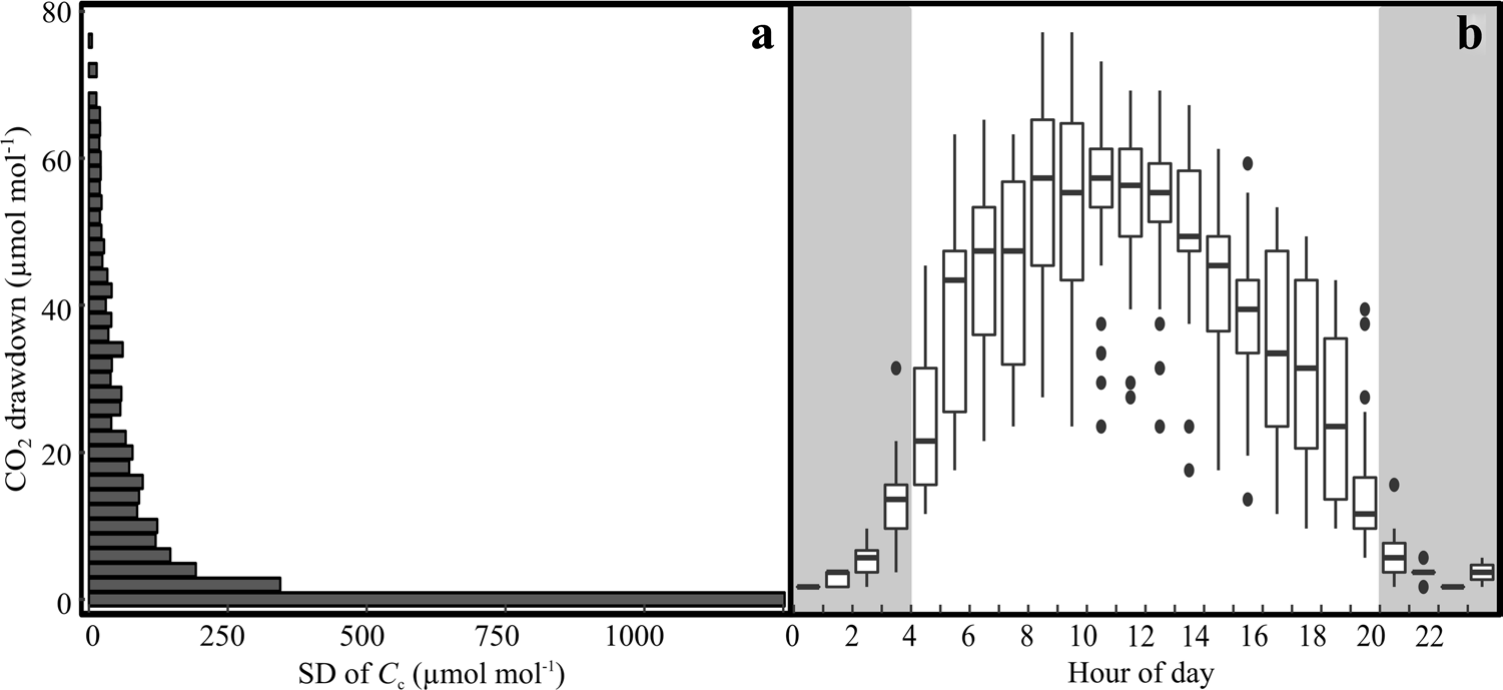
**a** Standard deviation of the CO_2_ concentration in the chloroplast (*C*_c_) in relation to CO_2_ drawdown in the cuvette, defined as the difference between the atmospheric concentration (*C*_in_), and the concentration inside the cuvette (*C*_out_). The SD of *C*_c_ was estimated for every 2 µmol mol^−1^ change in the CO_2_ drawdown. The figure is based on non-filtered data. **b** The diurnal time course of the CO_2_ drawdown. The whiskers of the box-plots extend to 1.5 times the interquartile range. The grey area marks the time of day excluded from the analysis. The figure is based on non-filtered data

We evaluated how the magnitude of the net photosynthetic CO_2_ drawdown, calculated as *C*_in_−*C*_out_, affected our estimates of *C*_c_. This drawdown, together with instrument precision, determines the error associated with ∆^13^C measurements, which ultimately determines the error in *C*_c_ and *g*_m_ estimates (for a discussion see Ubierna et al. 2018). The concentration drop is evaluated with the parameter ζ = *C*_in_/(*C*_in_−*C*_out_). Pons et al. (2009) showed that the error associated with *g*_m_ estimates increased when ζ was large and the instrument precision was low. We likewise found that *C*_c_ became exponentially more variable as the CO_2_ drawdown in the cuvette decreased below 20 μmol CO_2_ mol^−1^ (Fig. 1a). Assuming an ambient CO_2_ concentration of 400 ppm, a drawdown of 20 μmol CO_2_ mol^−1^ corresponds to ζ = 20 (= 400/(400–380)). In this case, and with an instrument precision of 0.06‰ the error associated with ∆^13^C measurements was 1.7‰ (= $\sqrt 2 \cdot \zeta \cdot {\text{Precision)}}$. This low drawdown and large error mainly occurred during early mornings and evenings, so that it was not possible to acquire reasonable estimates before 04:00 and after 20:00 (Fig. 1b). For most of the day, the observed drawdown was substantially greater than 20 μmol CO_2_ mol^−1^ which resulted in ζ < 10 and associated errors in ∆_o_ < 0.8‰ (Fig. 1b).

### Comparison of models to estimate water-use efficiency from ∆^13^C

Instantaneous WUE can be derived from ∆^13^C (WUE_∆_) or from gas exchange (WUE_G_) measurements as (Seibt et al. 2008; Hu et al. 2010; Wang et al. 2014; Klein et al. 2015; Guerrieri et al. 2016):

3 $$ WUE_{\text{t}} = \left\{ {\begin{array}{ll} {a)\, {\text{from}}\, \Delta^{ 1 3} C \to WUE_{\Delta } = \frac{{(C_{\text{out}} - C_{\text{i}} ) * VPD}}{1.6}} \\ {b)\,{\text{from gas exchange }} \to WUE_{\text{G}} = \frac{{A_{\text{net}} }}{E}} \\ \end{array} } \right\} $$

where *C*_i_ is solved from a theoretical ∆^13^C model.

We considered three models for ∆^13^C, which resulted in three estimations of WUE_∆_. First we estimated *C*_i_ from the simple model by Farquhar et al. (1982), as

4 $$ C_{\text{i}} = C_{\text{out}} * \frac{{\Delta_{\text{o}} - a_{\text{s}} }}{{\bar{b} - a_{\text{s}} }} $$

where $\bar{b}$ is taken as 27‰, a standard value for C_3_ plants, that was derived empirically from relationships between δ^13^C of leaf bulk material and *C*_i_/*C*_a_ values (Farquhar et al. 1982; Cernusak et al.^2013^; Ubierna and Farquhar 2014). This model does not account specifically for the dependency of ∆^13^C on *g*_m_, *R*_d_ or photorespiration (*R*_p_); instead it includes these effects empirically within $\bar{b}$, which is often sufficient in practice (Cernusak et al. 2013; Bloomfield et al. 2019). Second, we estimated *C*_i_ from a model proposed by Seibt et al. (2008), subsequently referred to as the partial model, as

5 $$ C_{{\text{i}}}  = C_{{{\text{out}}}} *\frac{{\Delta _{{\text{o}}}  - a_{{\text{s}}}  + \left( {b - a_{{\text{m}}} } \right)\frac{{g_{{\text{s}}} }}{{1.6*g_{{\text{m}}} }} + f\frac{{\Gamma ^{*} }}{{C_{{{\text{out}}}} }}}}{{b - a_{{\text{s}}}  + \left( {b - a_{{\text{m}}} } \right)\frac{{g_{{\text{s}}} }}{{1.6*g_{{\text{m}}} }}}} $$

where Γ* is the CO_2_ compensation point, derived from an Arrhenius function (Bernacchi et al. 2001; Medlyn et al. 2002 Eq. 12). This model accounts explicitly for the effect of *g*_m_ and *R*_p_ and assumes negligible effect of *R*_d_. Finally, from the comprehensive model of Farquhar and Cernusak (2012) *C*_i_ can be solved as (see supplemental materials in Cernusak et al. 2018)

6 $$ C_{\text{i}} = \frac{{- {\text{II}} \pm \sqrt {{\text{II}}^{2} - 4{\text{I}}*{\text{III}}}}}{{2{\text{I}}}} $$

where equations I, II and III are given in the supplementary materials (Eqn S9–S11). This model accounts explicitly for *g*_m_, *R*_p_ and *R*_d_, and it includes a correction for the ternary effect.

To avoid circularity, we divided our data set into two parts. We used 4 days’ data to estimate a mean value of *g*_m_ (0.29 mol CO_2_ m^−2^ s^−1^ bar^−1^). Subsequently, this mean *g*_m_ was used on the remaining 5 days’ data to estimate *C*_i_ from ∆^13^C (Eqs. 4, 5 and 6) and WUE (Eq. 3). Note that the 4-day mean *g*_m_ was slightly different from the mean for all 9 days together, which was 0.31 mol CO_2_ m^−2^ s^−1^ bar^−1^. For the comprehensive model, besides using a constant mean *g*_m_, we also performed the calculations using either a constant *g*_m_/*g*_s_ (2.9) or infinite *g*_m_ and compared these estimates as well to WUE_G_.

### Data analysis

A statistical filter was applied to the dataset to discard outliers in *C*_i_−*C*_c_ and *g*_m_. Any data point outside the range of mean ± 3 SD was considered an outlier and removed. This filter removed 4.5% of the data. Despite the filtering, some few negative conductances remained. Although they are not theoretically possible, we retained them in the analysis because they represent the tails of the statistical distributions and they influenced the means. The sole exception was when we analysed the dependency of net photosynthetic rates on the conductances. In this one analysis, the negative values were deleted. The four cuvettes were treated as biological replicates, from which the mean hourly values were used for further analysis. Regression analysis was used to evaluate diurnal patterns. This included linear, polynomial, and nonlinear regression, as deemed appropriate. Correlations were treated as significant for *p* ≤ 0.05. All variability is given as standard error, unless stated otherwise. All statistical analyses were performed using the base package of R (version 3.3.2).

## Results

### Diurnal trends of *g*_s_ and *g*_m_

**Fig. 2:**
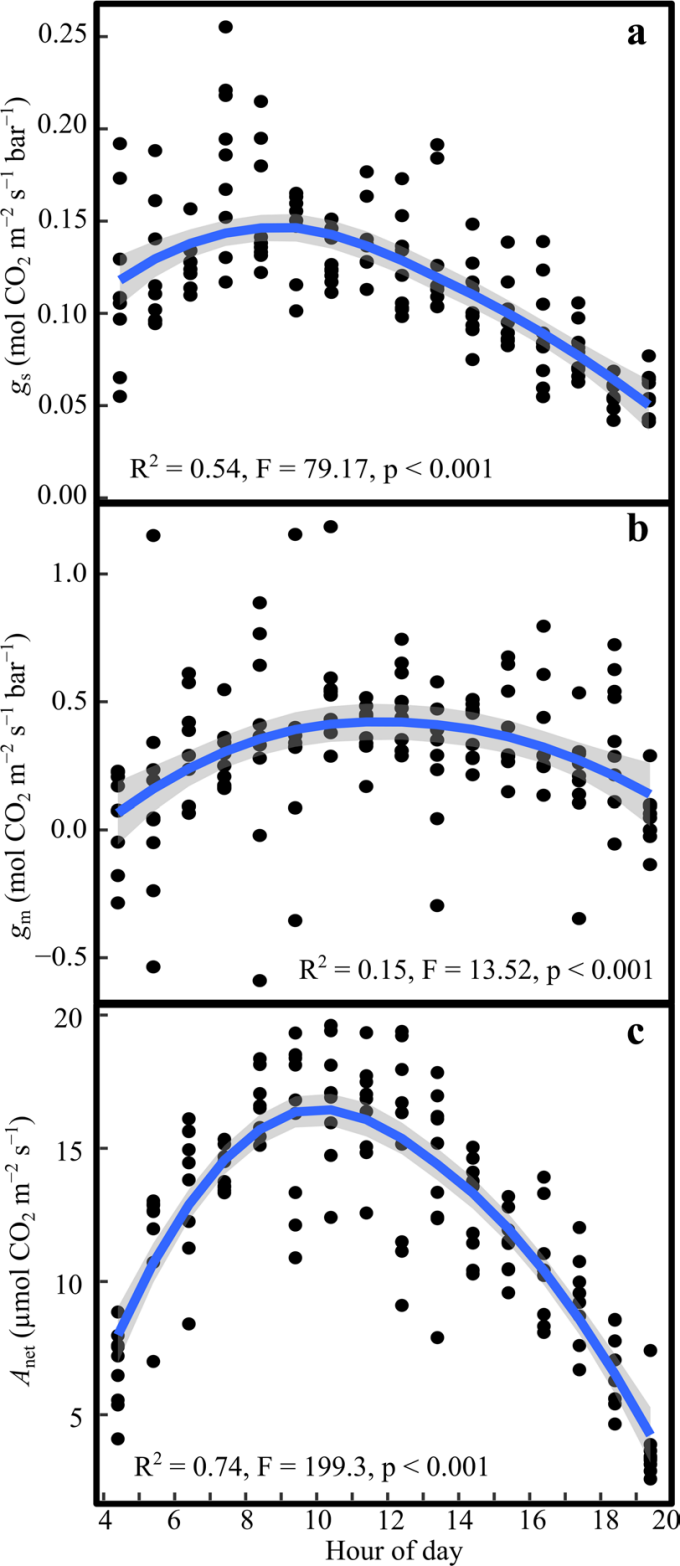
Diurnal variation in **a** stomatal conductance (*g*_s_), **b** mesophyll conductance (*g*_m_), and **c** net photosynthesis (*A*_net_). The points represent the cuvette means (*n* = 4) for each hour and day. The blue line is the second order polynomial fit to the data and the shaded grey area is the standard error of the fit

We evaluated the diurnal trends in stomatal and mesophyll conductance, and in their ratio. Mean *g*_s_ was 0.115 (SE = 0.002) mol CO_2_ m^−2^ s^−1^ bar^−1^. As expected, *g*_s_ showed a significant diurnal pattern (*F* = 79.17, *p* < 0.001), with peak values between 09:00 and 10:00, and decreased thereafter (Fig. 2a). We found a mean *g*_m_ value of 0.31 (SE = 0.02) mol CO_2_ m^−2^ s^−1^ bar^−1^. Furthermore *g*_m_ also had a significant diurnal pattern (*F* = 13.52, *p* < 0.001; Fig. 2b) with relatively stable mean values between 08:00 and 16:00 and lower values in the early morning and towards the evening. The mean for the unitless ratio *g*_m_/*g*_s_ was 2.67 (SE = 0.3); with a weak, but significant diurnal pattern (*F* = 3.9, *p* = 0.02).

### The relationship of *A*_net_ to *g*_s_ and *g*_m_

**Fig. 3:**
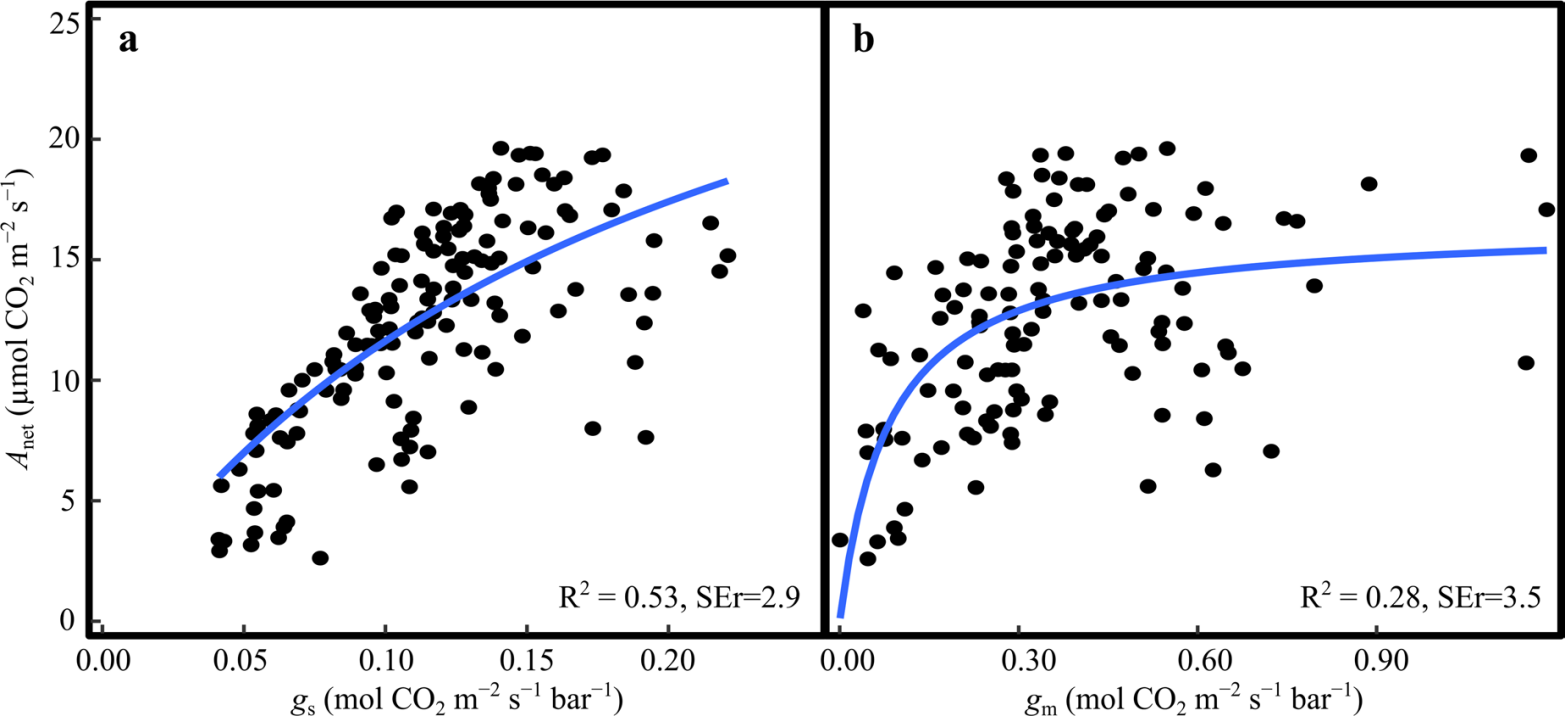
Relationship between net photosynthesis (*A*_net_) and **a** stomatal conductance (*g*_s_), and **b** mesophyll conductance (*g*_m_). The points represent the cuvette means (*n* = 4) for each hour and day. The blue line represents the asymptotic fit to the data

*A*_net_ followed a typical diurnal pattern, with highest rates between 08:00 and 12:00, with a mean of 16.2 (SE = 0.26) µmol CO_2_ m^−2^ s^−1^, and gradually declining rates in the afternoon (Fig. 2c). We found a significant asymptotic relationship between *A*_net_ and *g*_s_ (*p* < 0.001, *R*^2^ = 0.53, Fig. 3a). Similarly there was a significant asymptotic relationship between *A*_net_ and *g*_m_ (*p* < 0.001, *R*^2^ = 0.28, Fig. 3b).

### Relationship of *g*_s_ and *g*_m_ to VPD

**Fig. 4:**
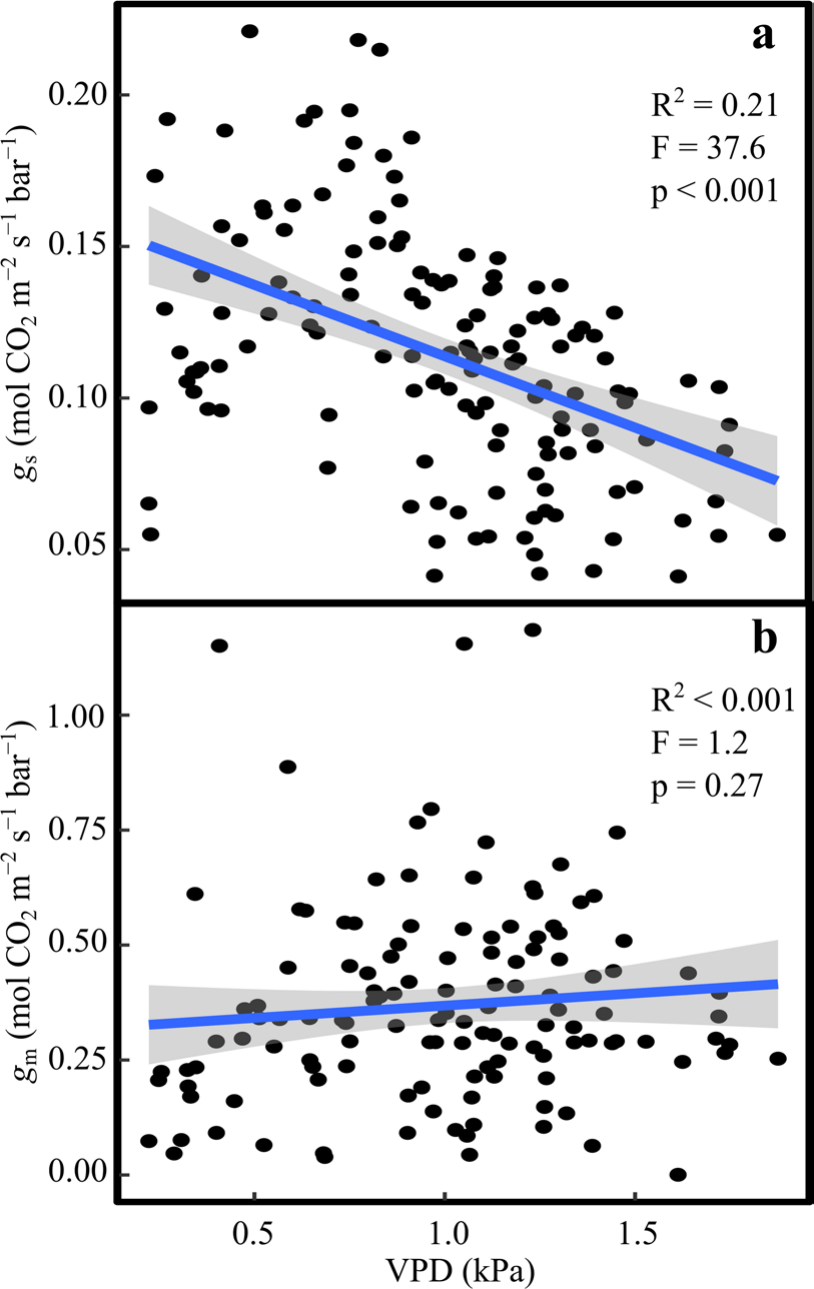
Response of **a** stomatal (*g*_s_), and **b** mesophyll (*g*_m_) conductance to vapour pressure deficit (*VPD*). The points represent the cuvette means (*n* = 4) for each hour and day. The blue line represents the regression fit to the data and the shaded grey area is the standard error of the fit

The average hourly VPD varied from 0.26 kPa to 1.82 kPa during the day, with sharp increase during the mornings until about 12:00 (Fig S4). We found a significant linear relationship between *g*_s_ and *VPD* (*F* = 37.6, *p* < 0.001, *R*^2^ = 0.26) (Fig. 4a) but no relationship between *g*_m_ and VPD (*F* = 1.12, *p* = 0.3, *R*^2^ = 0.001) (Fig. 4b).

### Contrasting estimates of WUE from ∆^13^C

**Fig. 5:**
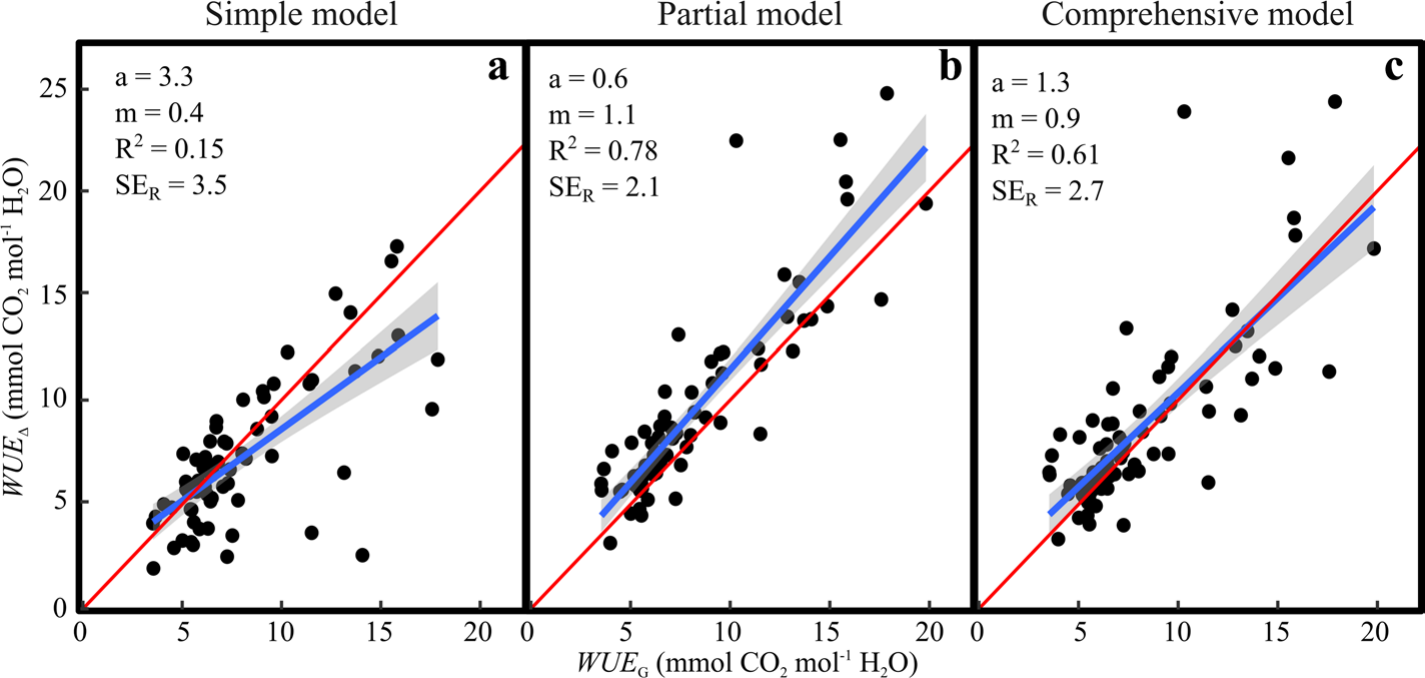
Water-use efficiency calculated from ∆^13^C data (WUE_∆_) using **a** the simple model **b** the partial model, and **c** comprehensive model presented here. The results are compared to water-use efficiency calculated from continuous gas exchange data (WUE_G_). In model **b** and **c** we assume a constant vale for *g*_m_ (0.29 mol CO_2_ m^−2^ s^−1^ bar^−1^). The points represent the cuvette means (*n* = 4) for each hour and day. The blue line is the linear fit to the data, the shaded grey area is the standard error of the fit, and m is the slope of the fit. The red line represents the theoretical 1:1 fit for comparison

We compared the performance of the three models to estimate WUE from *∆*^13^C (WUE_∆_) against direct measurements of WUE as *A*_net_*/E* by the gas exchange system (WUE_G_). Theoretically WUE_∆_ and WUE_G_ should be identical with an ideal fit, where slope *m* = 1 and intercept *a* = 0. Using the simple model resulted in a poor fit to our data (Fig. 5a). Further analysis revealed that this model could not predict WUE_G_ in the early hours (04:00–08:00), but fit the data well between 08:00 and 20:00 (*a* = − 0.4, *m* = 1.0, *R*^2^ = 0.69, Fig S5). The partial model consistently overestimated WUE_G_ by ca. 15% with no diurnal pattern (Fig. 5b). The comprehensive model matched the data well on average (Fig. 5c), but had a slight tendency to overestimate WUE_G_ in the low range and underestimate it in the high range. Furthermore, it introduced more variability into the estimates compared to the partial model, with a residual standard error (SEr) of 2.7 mmol CO_2_ mol H_2_O^−1^ (*R*^2^ = 0.61) compared to 2.1 mmol CO_2_ mol H_2_O^−1^ (*R*^2^ = 0.78). In the comprehensive model, representing *g*_m_ as a constant ratio to *g*_s_ overestimated WUE_G_ by ca. 9% compared to observations (*a* = 0.7, *m* = 1.05, Fig. S6a). Assuming infinite *g*_m_ resulted in a poor fit to observed data (*a* = 0.7, *m* = 1.5, Fig. S6b) and an overestimation of WUE_G_ by 49%.

## Discussion

Here, we report the first *g*_m_ estimates for mature *Pinus sylvestris*, one of the most widespread coniferous species of the northern hemisphere. Our mean value (0.31, SE = 0.02 mol m^−2^ s^−1^ bar^−1^) is somewhat higher than previously reported for other *Pinus* species (Flexas et al. 2008), but it is within the range of values reported for various conifers (Table 1) (De Lucia et al. 2003; Flexas et al. 2008; Bown et al. 2009; Bickford et al. 2010; Han 2011; Maseyk et al. 2011; Ubierna and Marshall 2011; Veromann-Jürgenson et al. 2017). Some of this variation may be due to differences in the methods used (Flexas et al. 2008). In particular, the “variable *J* method”, which uses simultaneous measurements of gas exchange and chlorophyll fluorescence to infer *g*_m_, generally yields lower *g*_m_ values than do isotopic techniques. If we exclude the “variable *J*” estimates from the list in Table 1, then our estimate of *g*_m_ matches the other values for conifers quite well. Furthermore, our estimates of δ^13^C of *A*_net_ were in the range reported previously (e.g. Wingate et al. 2010) (Fig. S7), and are the first based on measurements using the CRDS (cavity ring-down spectroscopy) technology. The good agreement encourages us to suggest that this method, which is less expensive than most alternatives, produces reliable δ^13^C measurements and is suitable for field applications. The CRDS was steady under the variable conditions of field-measurements, exemplified by the fact that we did not see any drift in the δ^13^C values of the reference gases during several weeks of continuous measurements (Fig. S3). Nevertheless, it was crucial to correct the data for the CRDS’ sensitivity to changing CO_2_ and H_2_O vapour concentrations. The sensitivity of isotope measurements to CO_2_ concentration is a known phenomenon and is commonly corrected for in other laser technologies, like in lead alloy tunable diode lasers (Ubierna et al. 2018). Furthermore, drying the gas before isotope analysis will avoid having to correct for H_2_O concentrations dependency, which is likely to improve measurement accuracy.

**Table 1:** Mesophyll conductance values reported for various coniferous species

Measurement method	Species	*g*_m_ mol m^−2^ s^−1^ bar^−1^**	References
Instantaneous ∆^13^C and gas exchange (CRDS)	*Pinus sylvestris*	0.33 ± 0.03	Current paper
Instantaneous ∆^13^C and gas exchange	*Pinus halepensis*	0.2–0.4	Maseyk et al. (2011)
Instantaneous ∆^13^C and gas exchange (TDL)	*Juniperus monosperma*	0.04–4.0	Bickford et al. (2009)
Instantaneous ∆^13^C and gas exchange	*Pseudotsuga menziesii*	0.14–0.20	Warren et al. (2003)
Phloem δ^13^C	*Pseudotsuga menziesii*	0.22 ± 0.11	Ubierna and Marshall (2011)
Phloem δ^13^C	*Thuja plicata*	0.09 ± 0.04	Ubierna and Marshall (2011)
Phloem δ^13^C	*Larix occidentalis*	0.41 ± 0.21	Ubierna and Marshall (2011)
Variable J method	*Pinus radiata*	0.153 ± 0.002	De Lucia et al. (2003)
Variable J method	*Pinus sylvestris*	0.124 ± 0.009	Veromann-Jürgensson et al. (2017)
Variable J method	*Pinus radiata*	0.16–0.2	Bown et al. (2009)
Variable J method	*Pinus densiflora*	0.107–0.250	Han (2011)
Various methods	*Pinus* spp.*	0.04–0.17	Flexas et al. (2008)

*Pinus lambertiana, *Pinus monticola*, *Pinus pinaster*, *Pinus radiata*

**If published values were presented as mol m^−2^ s^−1^, then we assumed barometric pressure of 1 bar and converted the units to mol m^−2^ s^−1^ bar^−1^

We observed an asynchronous reduction in *g*_m_ and *g*_s_ over the day (Fig. 2a, b). This may happen because *g*_s_ is under strong control by ambient *VPD* (Fig. 4a), whereas we found no correlation between *g*_m_ and *VPD* (Fig. 4b). The response of *g*_m_ to *VPD* has only been investigated in few studies, and with contrasting results. While Bongi and Loreto (1989) and Loucos et al. (2017) found a significant negative correlation between *g*_m_ and *VPD*, a study looking at the effect of air humidity and soil moisture (Warren 2008c) on *g*_s_ and *g*_m_ found a strong correlation of both conductances to soil moisture, while *VPD* only affected *g*_s_ and not *g*_m_. All studies involved different species, and higher *VPD* ranges (1–3 kPa and 1–2 kPa, respectively) compared to our study (0.23–1.82 kPa). These discrepancies highlight the need for further investigation including a wider range of *VPD* conditions for *Pinus sylvestris*.

We fitted an asymptotic relationship of *A*_net_ to *g*_s_ and *g*_m_. The asymptotic response agrees with theoretical expectations of CO_2_ saturation at high conductances. In a diurnal context, *A*_net_ was maintained at high rates until mid-day, despite declining *g*_s_ from mid-morning (Fig. 2). We suggest that high *g*_m_ helped to maintain *A*_net_ during the late morning, enabling high *C*_c_ and compensating for the decline of *g*_s_. This diurnal asynchrony between *g*_s_ and *g*_m_ is qualitatively similar to the observations by Theroux-Rancourt et al. (2014) on hybrid poplar cuttings exposed to soil drying over 12 days. They suggested based on daily measurements of *g*_s_ and *g*_m_ that a delayed *g*_m_ response reduced the decline in photosynthesis and enhanced WUE during the beginning of the drought treatment. Our finding suggests, that even within a diurnal context, the asynchronous response of *g*_m_ and *g*_s_ to environmental conditions has significant influence on *A*_net_ and presumably WUE.

We compared three models to estimate WUE from δ^13^C. We found that the simple model can estimate WUE well for most of the day. This model uses $\bar{b}$, an empirical value that accounts for the drop of concentration between *C*_i_ and *C*_c_, the different fractionations occurring during photosynthetic discrimination, as well as possible post-photosynthetic discrimination. Many studies have shown that $\bar{b}$ works well as an approximation (e.g. Farquhar et al. 1982; Seibt et al. 2008; Bloomfield et al. 2019). However, it performed poorly during the early morning, when WUE (Fig. S8), and especially δ^13^C of photosynthesis (Fig. S7) were more variable. This meant that it produced unreliable WUE estimates for 25% of the photosynthetically active period of the day. Our analysis clearly shows that a more complete model that accounts explicitly for the effects of photorespiration, or both photorespiration and daytime respiration, performs better under such variable conditions and provides more accurate estimates of WUE. The relatively high variability in the estimates highlight the need to further refine some of the model assumptions.

We have shown that it is critical to account for *g*_m_ in the estimation of WUE from ∆^13^C. This point has been made before (Seibt et al. 2008; Klein et al. 2015), but is still often neglected. Our data is yet an other example of WUE being overestimated if *g*_m_ is assumed to be infinite, and we show that assuming constant *g*_m_/*g*_s_ or constant *g*_m_ both yield better estimates than infinite *g*_m_. Estimating WUE from *g*_m_/*g*_s_ had the further advantage of accounting for some of the diurnal change in *g*_m_, resulting in a slope closer to 1 than when *g*_m_ was assumed constant. Nevertheless, this approach does not take into account the diurnality of *g*_m_/*g*_s_ itself, and neglects the fact that *g*_m_ is much less strongly correlated with VPD than *g*_s_.

The current study presents the first estimate of *g*_m_ for mature *Pinus sylvestris* trees, one of the most wide-ranging tree species in the world. Those estimates were derived with a CRDS/gas exchange system, which presents opportunities for simplifying the measurement of online ∆^13^C discrimination. The measurements were made continuously and in the field over several sunny days in the summer. The high temporal resolution of our data allowed us to evaluate diurnal trends in conductance in relation to *A*_net_, and test different models to estimate WUE form ∆^13^C. Our analysis revealed that the simple model to account for ^13^C discrimination worked well, but only under stable conditions, and that the comprehensive model has the potential to account for variable conditions and provide reliable estimates of *C*_i_ and WUE. We highlight the need for further work under a broader range of environmental conditions, and including seasonal phenology. Our *g*_m_ estimate provides a means of improving inferences of WUE from ∆^13^C and our continuous measurements provide a path forward to improve the modelling of *g*_m_ in the future.

## Electronic supplementary material

Below is the link to the electronic supplementary material.

Supplementary material 1 (DOCX 3156 kb)

## Abbreviations

*A*_net_: Net photosynthesis rate (µmol CO_2_ m^−2^ s^−1^)
∆_e_: Discrimination associated with respiration (‰)
∆_f_: Discrimination associated with photorespiration (‰)
∆_i_: Discrimination when *C*_i_ = *C*_c_ (‰)
∆_o_: Observed photosynthetic discrimination (‰)
*a*_m_: ^12^C/^13^C fractionation during dissolution and diffusion through water 1.8 (‰)
*a*_s_: ^12^C/^13^C fractionation during diffusion through air 4.4 (‰)
*b*: ^12^C/^13^C fractionation during carboxylation 29 (‰)
$\bar{b}$: ^12^C/^13^C net fractionation during carboxylation 27 (‰)
*C*_c_: CO_2_ concentration in the chloroplast (µmol mol^−1^)
*C*_i_: CO_2_ concentration in the intercellular space (µmol mol^−1^)
*C*_in_: CO_2_ concentration in the air entering the cuvette (µmol mol^−1^)
*C*_out_: CO_2_ concentration in the cuvette (µmol mol^−1^)
*E*: Transpiration rate (mmol H_2_O m^−2^ s^−1^)
*e*: Fractionation during respiration (‰)
*f*: Fractionation during photorespiration 16.2 (‰)
*g*_m_: Mesophyll conductance (mol CO_2_ m^−2^ s^−1^ bar^−1^)
*g*_s_: Stomatal conductance (mol CO_2_ m^−2^ s^−1^ bar^1^)
*R*_d_: Day time respiration rate (µmol CO_2_ m^−2^ s^−1^)
*R*_p_: Photorespiration rate (µmol CO_2_ m^−2^ s^−1^)
*t*: Ternary correction factor (–)
VPD: Vapour pressure deficit (kPa)
WUE: Water-use efficiency (mmol CO_2_ mol^−1^ H_2_O)
WUE_G_: WUE from gas exchange (mmol CO_2_ mol^−1^ H_2_O)
WUE_∆_: WUE calculated from *∆*^13^C (mmol CO_2_ mol^−1^ H_2_O)
Γ***: CO_2_ compensation point (µmol mol^−1^)

## Footnotes

1 In calculations based on on-line discrimination, we consider the CO_2_ concentration and the isotopic composition inside the cuvette (noted with the subscript ‘out’) as ‘ambient’ because they represent the immediate environment of the shoot or leaf. If the models presented here (Eqs. 1, 3, 4, 5) are to be applied elsewhere, *C*_out_ can be replaced with *C*_a_ and ^13^*C*_out_ can be replaced with ^13^*C*_a_.

## References

[CR1] Bernacchi C, Singsaas E, Pimentel C, Portis AR, Long SP (2001). Improved temperature response functions for models of Rubisco limited photosynthesis. Plant, Cell Environ.

[CR2] Bernacchi CJ, Portis AR, Nakano H, von Caemmerer S, Long SP (2002). Temperature response of mesophyll conductance. Implications for the determination of Rubisco enzyme kinetics and for limitations to photosynthesis in vivo. Plant Physiol.

[CR3] Bickford C, McDowell N, Erhardt E, Hanson D (2009). High-frequency field measurements of diurnal carbon isotope discrimination and internal conductance in a semi-arid species, *Juniperus monosperma*. Plant, Cell Environ.

[CR4] Bickford CP, Hanson DT, McDowell NG (2010). Influence of diurnal variation in mesophyll conductance on modelled 13C discrimination: results from a field study. J Exp Bot.

[CR67] Bloomfield KJ, Prentice IC, Cernusak LA, Eamus D, Medlyn BE, Rumman R, Wright IJ, Boer MM, Cale P, Cleverly J, Egerton JJ, Ellsworth DS, Evans BJ, Hayes LS, Hutchinson MF, Liddell MJ, Macfarlane C, Meyer WS, Togashi HF, Wardlaw T, Zhu L, Atkin OK (2019). The validity of optimal leaf traits modelled on environmental conditions. New Phytol.

[CR5] Bongi G, Loreto F (1989). Gas-exchange properties of salt-stressed olive (*Olea europea* L.) leaves. Plant Physiol.

[CR6] Bown HE, Watt MS, Mason EG, Clinton PW, Whitehead D (2009). The influence of nitrogen and phosphorus supply and genotype on mesophyll conductance limitations to photosynthesis in *Pinus radiata*. Tree Physiol.

[CR7] Bunce JA (2010). Variable responses of mesophyll conductance to substomatal carbon dioxide concentration in common bean and soybean. Photosynthetica.

[CR8] Campany CE, Tjoelker MG, von Caemmerer S, Duursma RA (2016). Coupled response of stomatal and mesophyll conductance to light enhances photosynthesis of shade leaves under sunflecks. Plant, Cell Environ.

[CR9] Cernusak LA, Ubierna N, Winter K, Holtum JAM, Marshall JD, Farquhar GD (2013). Environmental and physiological determinants of carbon isotope discrimination in terrestrial plants. New Phytol.

[CR10] Cernusak LA, Ubierna N, Jenkins MW, Garrity SR, Rahn T, Powers HH, Hanson DT, Sevanto S, Wong SC, McDowell NG, Farquhar GD (2018). Unsaturation of vapour pressure inside leaves of two conifer species. Sci Rep.

[CR11] De Lucia EH, Whitehead D, Clearwater MJ (2003). The relative limitation of photosynthesis by mesophyll conductance in co-occurring species in a temperate rainforest dominated by the conifer *Dacrydium cupressinum*. Funct Plant Biol.

[CR12] Dewar R, Mauranen A, Mäkelä A, Hölttä T, Medlyn B, Vesala T (2017). New insights into the covariation of stomatal, mesophyll and hydraulic conductances from optimization models incorporating nonstomatal limitations to photosynthesis. New Phytol.

[CR13] Douthe C, Dreyer E, Epron D, Warren CR (2011). Mesophyll conductance to CO_2_, assessed from online TDL-AS records of ^13^CO_2_ discrimination, displays small but significant short-term responses to CO_2_ and irradiance in Eucalyptus seedlings. J Exp Bot.

[CR14] Evans JR, von Caemmerer S (2013). Temperature response of carbon isotope discrimination and mesophyll conductance in tobacco. Plant, Cell Environ.

[CR15] Evans J, Sharkey T, Berry J, Farquhar G (1986). Carbon isotope discrimination measured concurrently with gas exchange to investigate CO_2_ Diffusion in Leaves of higher plants. Funct Plant Biol.

[CR16] Farquhar GD, Cernusak LA (2012). Ternary effects on the gas exchange of isotopologues of carbon dioxide. Plant, Cell Environ.

[CR17] Farquhar GD, von Caemmerer S, Berry JA (1980). A biochemical model of photosynthetic CO_2_ assimilation in leaves of C3 species. Planta.

[CR18] Farquhar GD, Ball MC, von Caemmerer S, Roksandic Z (1982). Effect of salinity and humidity on δ^13^C value of halophytes-evidence for diffusional isotope fractionation determined by the ratio of intercellular/atmospheric partial pressure of CO_2_ under different environmental conditions. Oecologia.

[CR19] Flexas J, Diaz-Espejo A, Galmés J, Kaldenhoff R, Medrano H, Ribas-Carbo M (2007). Rapid variations of mesophyll conductance in response to changes in CO_2_ concentration around leaves. Plant, Cell Environ.

[CR20] Flexas J, Ribas-Carbo M, Diaz-Espejo A, Galmés J, Medrano H (2008). Mesophyll conductance to CO_2_: current knowledge and future prospects. Plant, Cell Environ.

[CR21] Flexas J, Galmés J, Gallé A, Gulias J, Pou A, Ribas-Carbo M, Tomas M, Medrano H (2010). Improving water use efficiency in grapevines: potential physiological targets for biotechnological improvement. Aust J Grape Wine Res.

[CR22] Flexas J, Barbour MM, Brendel O, Cabrera HM, Carriquí M, Díaz-Espejo A, Douthe C, Dreyer E, Ferrio JP, Gago J, Gallé A, Galmés J, Kodama N, Medrano H, Niinemets Ü, Peguero-Pina JJ, Pou A, Ribas-Carbó M, Tomás M, Tosens T, Warren CR (2012). Mesophyll diffusion conductance to CO_2_: an unappreciated central player in photosynthesis. Plant Sci.

[CR23] Galmés J, Conesa MA, Ochogavia JM, Perdomo JA, Francis DM, Ribas-Carbo M, Savé R, Flexas J, Medrano H, Cifre J (2011). Physiological and morphological adaptations in relation to water use efficiency in Mediterranean accessions of *Solanum lycopersicum*. Plant, Cell Environ.

[CR24] Guerrieri R, Lepine L, Asbjornsen H, Xiao J, Ollinger SV (2016). Evapotranspiration and water use efficiency in relation to climate and canopy nitrogen in U.S. forests. J Geophys Res.

[CR25] Han Q (2011). Height-related decreases in mesophyll conductance, leaf photosynthesis and compensating adjustments associated with leaf nitrogen concentrations in *Pinus densiflora*. Tree Physiol.

[CR26] Hasselquist NJ, Metcalfe DB, Högberg P (2012). Contrasting effects of low and high nitrogen additions on soil CO_2_ flux components and ectomycorrhizal fungal sporocarp production in a boreal forest. Glob Change Biol.

[CR27] Hassiotou F, Ludwig M, Renton M, Veneklaas EJ, Evans JR (2009). Influence of leaf dry mass per area, CO_2_, and irradiance on mesophyll conductance in sclerophylls. J Exp Bot.

[CR28] Hu J, Moore DJP, Riveros-Iregui DA, Burns SP, Monson RK (2010). Modeling whole-tree carbon assimilation rate using observed transpiration rates and needle sugar carbon isotope ratios. New Phytol.

[CR29] Keenan TF, Hollinger DY, Bohrer G, Dragoni D, Munger JW, Schmid HP, Richardson AD (2013). Increase in forest water-use efficiency as atmospheric carbon dioxide concentrations rise. Nature.

[CR30] Klein T, Rotenberg E, Tatarinov F, Yakir D (2015). Association between sap flow-derived and eddy covariance-derived measurements of forest canopy CO_2_ uptake. New Phytol.

[CR31] Lim H, Oren R, Palmroth S, Tor-ngern P, Mörling T, Näsholm T, Lundmark T, Helmisaari HS, Leppälammi-Kujansuu J, Linder S (2015). Inter-annual variability of precipitation constrains the production response of boreal *Pinus sylvestris* to nitrogen fertilization. For Ecol Manag.

[CR32] Loreto F, Tsonev T, Centritto M (2009). The impact of blue light on leaf mesophyll conductance. J Exp Bot.

[CR33] Loucos K, Simonin K, Barbour M (2017). Leaf hydraulic conductance and mesophyll conductance are not closely related within a single species. Plant, Cell Environ.

[CR34] Marshall JD, Monserud RA (1996). Homeostatic gas-exchange parameters inferred from ^13^C/^12^C in tree rings of conifers. Oecologia.

[CR35] Marshall JD, Waring RH (1984). Conifers and broadleaf species: stomatal sensitivity differs in western Oregon. Can J For Res.

[CR36] Maseyk K, Hemming D, Angert A, Leavitt SW, Yakir D (2011). Increase in water-use efficiency and underlying processes in pine forests across a precipitation gradient in the dry Mediterranean region over the past 30 years. Oecologia.

[CR37] Medlyn B, Dreyer E, Ellsworth D, Forstreuter M, Harley P, Kirschbaum M, Roux X, Montpied P, Strassemeyer J, Walcroft A, Wang K, Loustau D (2002). Temperature response of parameters of a biochemically based model of photosynthesis. II. A review of experimental data. Plant, Cell Environ.

[CR38] Medlyn B, Duursma R, Eamus D, Ellsworth D, Prentice I, Barton C, Crous K, Angelis P, Fremman M, Wingate L (2011). Reconciling the optimal and empirical approaches to modelling stomatal conductance. Glob Change Biol.

[CR39] Oren R, Sperry JS, Katul GG, Pataki DE, Ewers BE, Phillips N, Schäfer KV (1999). Survey and synthesis of intra-and interspecific variation in stomatal sensitivity to vapour pressure deficit. Plant, Cell Environ.

[CR40] Pons TL, Flexas J, von Caemmerer S, Evans JR, Genty B, Ribas-Carbo M, Brugnoli E (2009). Estimating mesophyll conductance to CO_2_: methodology, potential errors, and recommendations. J Exp Bot.

[CR41] Prentice IC, Dong N, Gleason SM, Maire V, Wright IJ (2014). Balancing the costs of carbon gain and water transport: testing a new theoretical framework for plant functional ecology. Ecol Lett.

[CR42] Rogers A, Medlyn BE, Dukes JS, Bonan G, von Caemmerer S, Dietze MC, Kattge J, Leakey ADB, Mercado LM, Niinemets Ü, Prentice CI, Serbin SP, Sitch S, Way DA, Zaehle S (2017). A roadmap for improving the representation of photosynthesis in Earth system models. New Phytol.

[CR43] Seibt U, Rajabi A, Griffiths H, Berry JA (2008). Carbon isotopes and water use efficiency: sense and sensitivity. Oecologia.

[CR44] Tarvainen L, Lutz M, Räntfors M, Näsholm T, Wallin G (2016). Increased needle nitrogen contents did not improve shoot photosynthetic performance of mature nitrogen-poor Scots pine trees. Front Plant Sci.

[CR45] Tarvainen L, Wallin G, Lim H, Linder S, Oren R, Ottosson Löfvenius M, Räntfors M, Tor-ngern P, Marshall JD (2018). Photosynthetic re-fixation varies along stems and reduces CO_2_ efflux in mature boreal *Pinus sylvestris* trees. Tree Physiol.

[CR46] Tazoe Y, von Caemmerer S, Estavillo GM, Evans JR (2011). Using tunable diode laser spectroscopy to measure carbon isotope discrimination and mesophyll conductance to CO_2_ diffusion dynamically at different CO_2_ concentrations. Plant, Cell Environ.

[CR47] Théroux-Rancourt G, Éthier G, Pepin S (2014). Threshold response of mesophyll CO_2_ conductance to leaf hydraulics in highly transpiring hybrid poplar clones exposed to soil drying. J Exp Bot.

[CR48] Tholen D, Boom C, Noguchi K, Ueda S, Katase T, Terashima I (2008). The chloroplast avoidance response decreases internal conductance to CO_2_ diffusion in *Arabidopsis thaliana* leaves. Plant, Cell Environ.

[CR49] Ubierna N, Farquhar GD (2014). Advances in measurements and models of photosynthetic carbon isotope discrimination in C3 plants. Plant, Cell Environ.

[CR50] Ubierna N, Marshall JD (2011). Estimation of canopy average mesophyll conductance using δ^13^C of phloem contents. Plant, Cell Environ.

[CR51] Ubierna N, Holloway-Phillips MM, Farquhar GD, Covshoff S (2018). Using stable carbon isotopes to study C3 and C4 photosynthesis: MODELS and calculations. Photosynthesis. Methods in molecular biology.

[CR52] Uddling J, Wallin G (2012). Interacting effects of elevated CO_2_ and weather variability on photosynthesis of mature boreal Norway spruce agree with biochemical model predictions. Tree Physiol.

[CR53] Veromann-Jürgenson LL, Tosens T, Laanisto L, Niinemets Ü (2017). Extremely thick cell walls and low mesophyll conductance: welcome to the world of ancient living!. J Exp Bot.

[CR54] Voelker S, Brooks R, Meinzer F, Anderson R, Bader M, Battipaglia G, Becklin K, Beerling D, Bert D, Betancourt J, Dawson TE, Domec J-C, Guyette R, Koerner C, Leavitt SW, Linder S, Marshall JD, Mildner M, Ogée J, Panyushkina I, Plumpton H, Pregitzer K, Saurer M, Smith A, Siegwolf R, Stambaugh M, Talhelm A, Tardif J, Van de Water P, Ward J, Wingate L (2016). A dynamic leaf gas-exchange strategy is conserved in woody plants under changing ambient CO_2_: evidence from carbon isotope discrimination in paleo and CO_2_ enrichment studies. Glob Change Biol.

[CR55] Von Caemmerer S (2000). Biochemical models of leaf photosynthesis. Techniques in plant science.

[CR56] Vrábl D, Vašková M, Hronková M, Flexas J, Šantrůček J (2009). Mesophyll conductance to CO_2_ transport estimated by two independent methods: effect of variable CO_2_ concentration and abscisic acid. J Exp Bot.

[CR57] Wallin G, Linder S, Lindroth A, Räntfors M, Flemberg S, Grelle A (2001). Carbon dioxide exchange in Norway spruce at the shoot, tree and ecosystem scale. Tree Physiol.

[CR58] Wang H, Zhao P, Zou LL, McCarthy HR, Zeng XP, Ni GY, Rao XQ (2014). CO_2_ uptake of a mature Acacia mangium plantation estimated from sap flow measurements and stable carbon isotope discrimination. Biogeosciences.

[CR59] Warren CR (2008). Stand aside stomata, another actor deserves centre stage: the forgotten role of the internal conductance to CO_2_ transfer. J Exp Bot.

[CR60] Warren CR (2008). Does growth temperature affect the temperature responses of photosynthesis and internal conductance to CO_2_? A test with *Eucalyptus regnans*. Tree Physiol.

[CR61] Warren CR (2008). Soil water deficits decrease the internal conductance to CO_2_ transfer but atmospheric water deficits do not. J Exp Bot.

[CR62] Warren CR, Ethier GJ, Livingston NJ, Grant NJ, Turpin DH, Harrison DL, Black TA (2003). Transfer conductance in second growth Douglas-fir (*Pseudotsuga menziesii* (Mirb.)Franco) canopies. Plant, Cell Environ.

[CR63] Wei L, Marshall JD, Link TE, Kavanagh KL, Du E, Pangle RE, Gag PJ, Ubierna N (2014). A new δ^13^C submodel for 3-PG. Plant, Cell Environ.

[CR64] Wingate L, Seibt U, Moncrieff J, Jarvis P, Lloyd J (2007). Variations in ^13^C discrimination during CO_2_ exchange by *Picea sitchensis* branches in the field. Plant, Cell Environ.

[CR65] Wingate L, Ogée J, Burlett R, Bosc A, Devaux M, Grace J, Loustau D, Gessler A (2010). Photosynthetic carbon isotope discrimination and its relationship to the carbon isotope signals of stem, soil and ecosystem respiration. New Phytol.

[CR66] Yamori W, Noguchi K, Hanba YT, Terashima I (2006). Effects of internal conductance on the temperature dependence of the photosynthetic rate in spinach leaves from contrasting growth temperatures. Plant Cell Physiol.

