# Supplementary material for: Diurnal variation in mesophyll conductance and its influence on modelled water-use efficiency in a mature boreal Pinus sylvestris stand
Source: Photosynth Res. 2019 May 23;141(1):53–63. doi: 10.1007/s11120-019-00645-6 (PMC6612512; doi:10.1007/s11120-019-00645-6)
Supplement: Supplementary file 1 — Supplementary material 1 (DOCX 3156 kb) [file 11120_2019_645_MOESM1_ESM.docx]

**Diurnal variation in mesophyll conductance and its influence on modelled water-use efficiency in a mature boreal *Pinus sylvestris* stand**

Zsofia R. Stangl^1^, Lasse Tarvainen^1,2^, Göran Wallin^2^, Nerea Ubierna^3^, Mats Räntfors^2^, John D. Marshall^1*^

1. Department of Forest Ecology and Management, Swedish University of Agricultural Sciences, Umeå, Sweden

2. Department of Biological and Environmental Sciences, University of Gothenburg, Gothenburg, Sweden

3. Research School of Biology, The Australian National University, Canberra, ACT, Australia

*Corresponding author: john.marshall@slu.se; +46 72 248 0477

**Supplementary Figures**

**Fig. S1** CRDS (Picarro G2131-i) sensitivity to CO_2_ concentration

Change in δ^13^C values by CO_2_ concentration. The dots represent one-minute means of individual measurements, and the regression was fitted with a three-parameter asymptotic model:

$$y = a + \frac{d-c}{1+\frac{e}{x}}$$

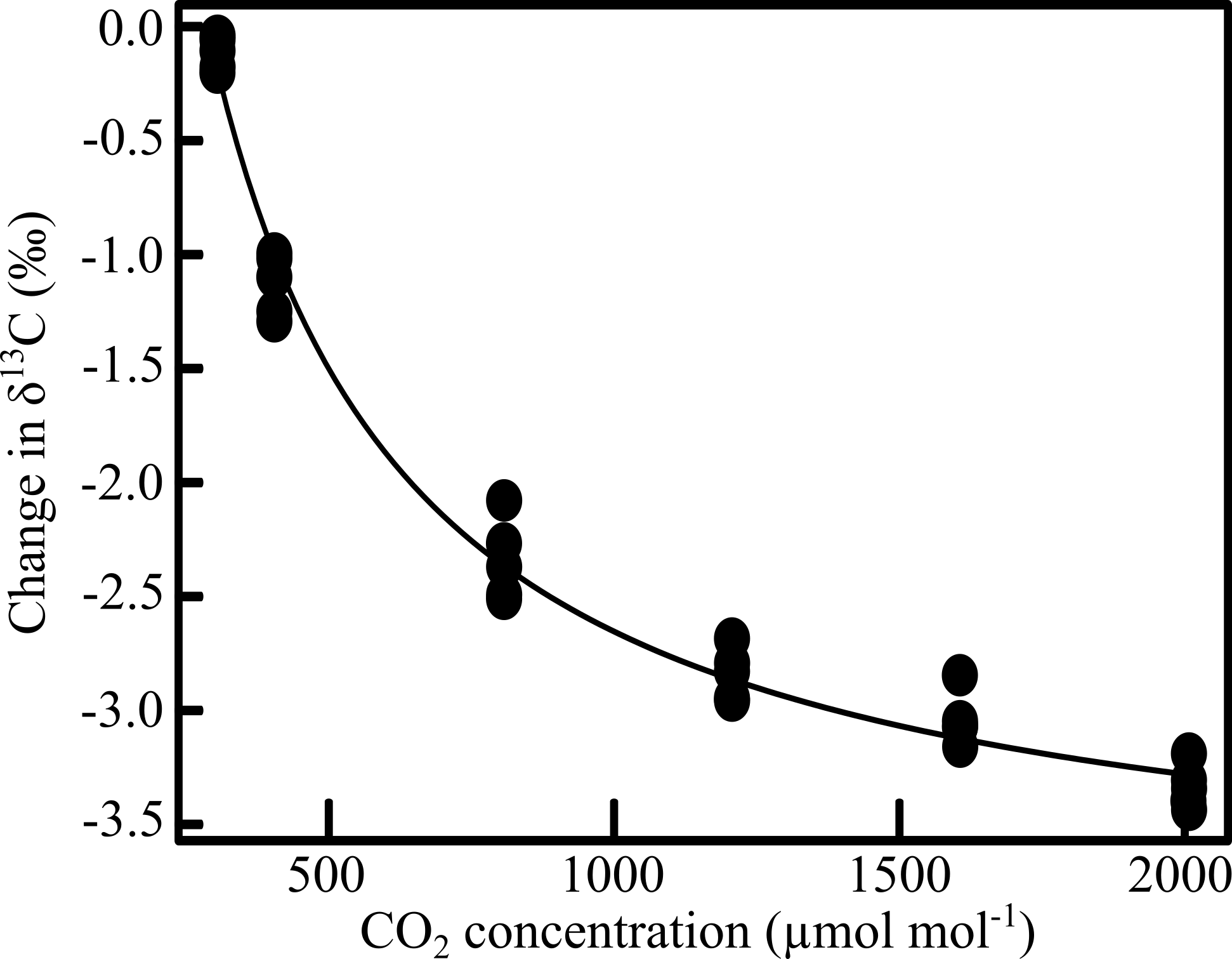


**Fig.S2** CRDS (Picarro G2131-i) sensitivity to H_2_O vapour concentration

Change in δ^13^C values by H_2_O_v_ concentration. The dots represent one-minute means of individual measurements, and the line is the linear fit to the data.


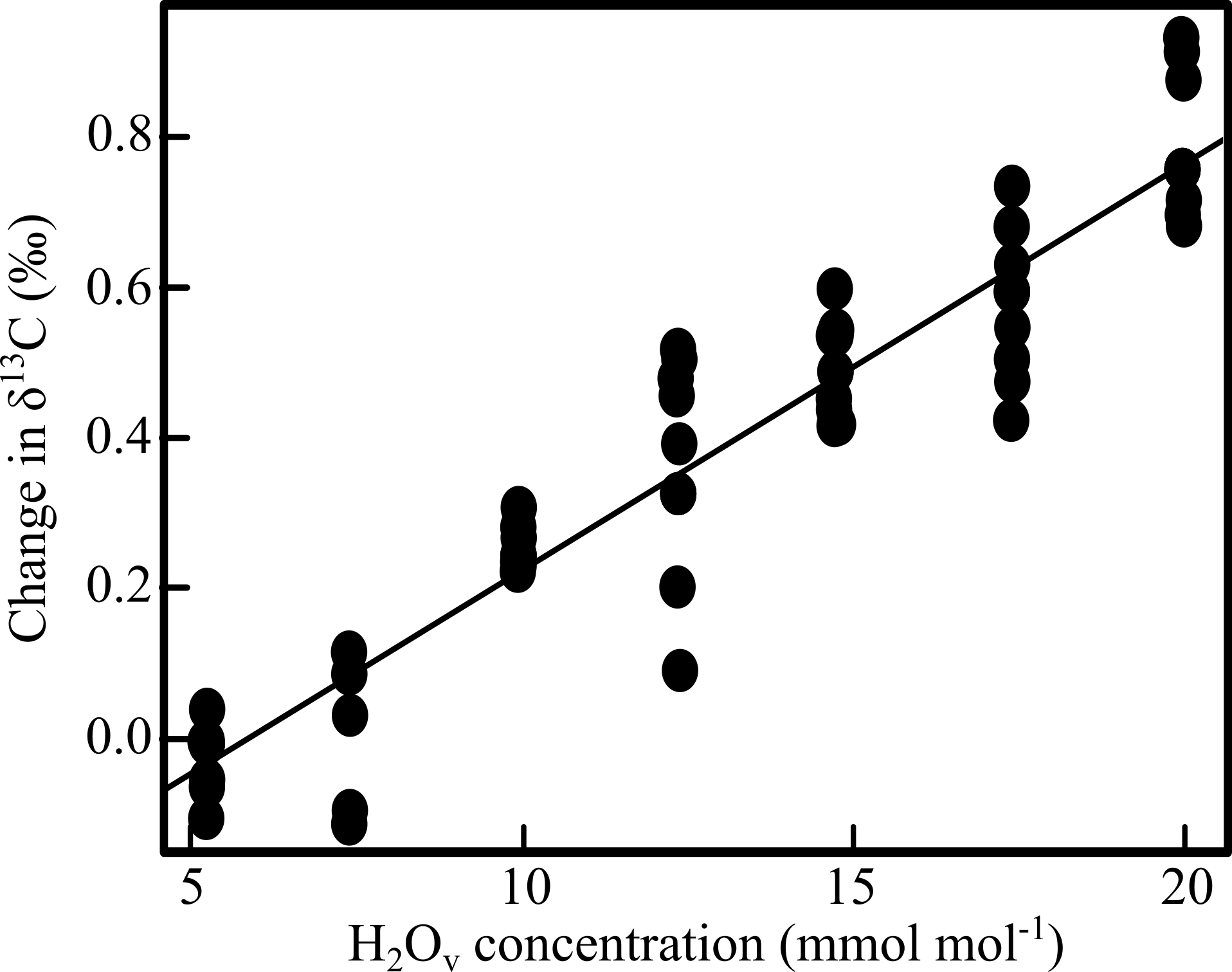


**Fig. S3** CRDS calibration data from May to June.

Calibration of the δ^13^C values with two reference gases: a) -4.14‰ and b) -32.36‰. The boxplots represent raw data, at approximately 1 Hz, from 5-minute calibration intervals. The boxes show the inner quartiles and the whiskers extend up to 1.5 times the interquartile range. Note the differing scale of the y-axis, which was adjusted in each plot for better resolution.

**
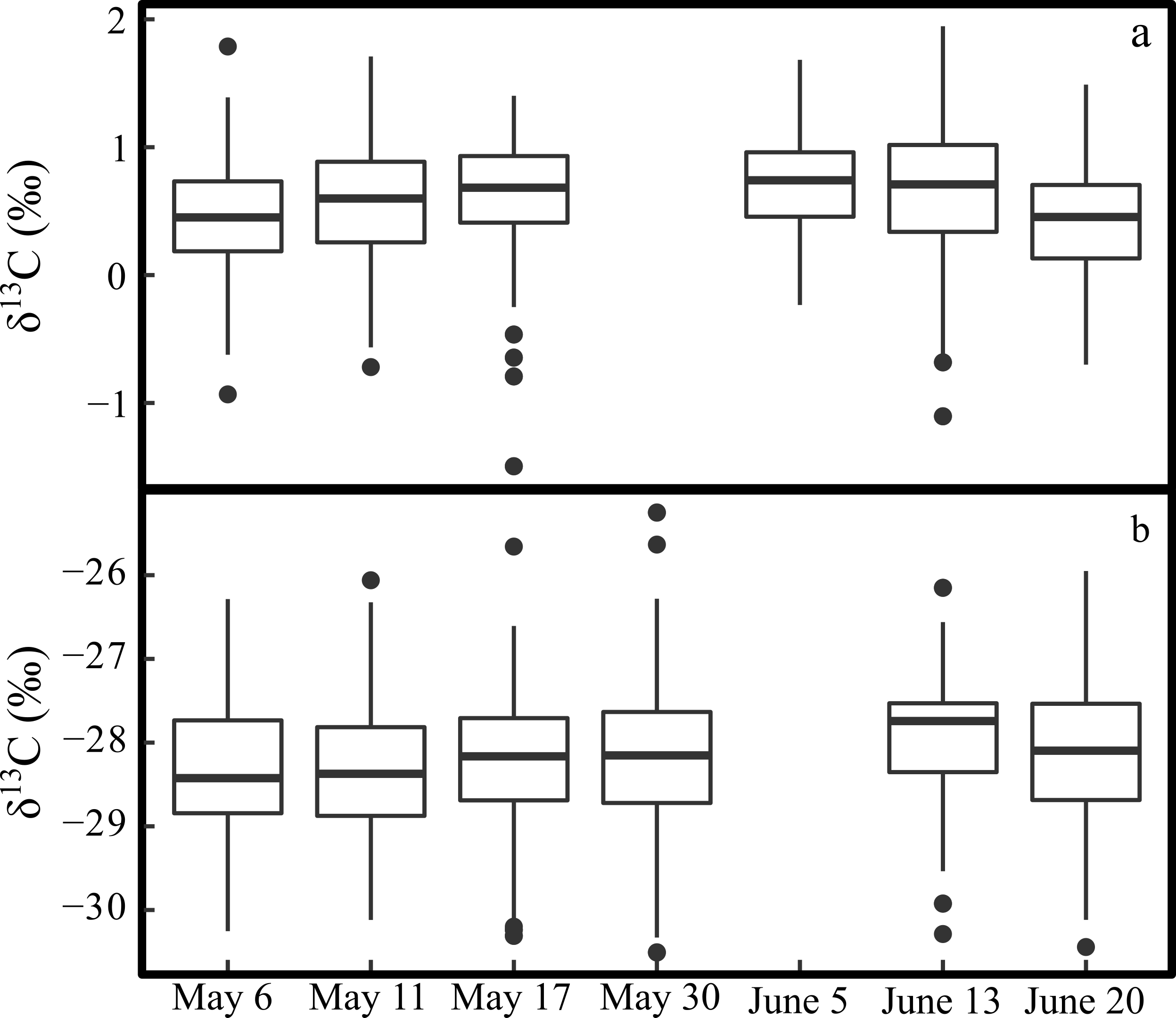
**

**Fig. S4** Diurnal variation in Vapour pressure deficit (VPD)

The points represent the cuvette means (*n* = 4) for each hour and day during the measurement period. The blue line is the second order polynomial fit to the data and the shaded grey area is the standard error of the fit.

**
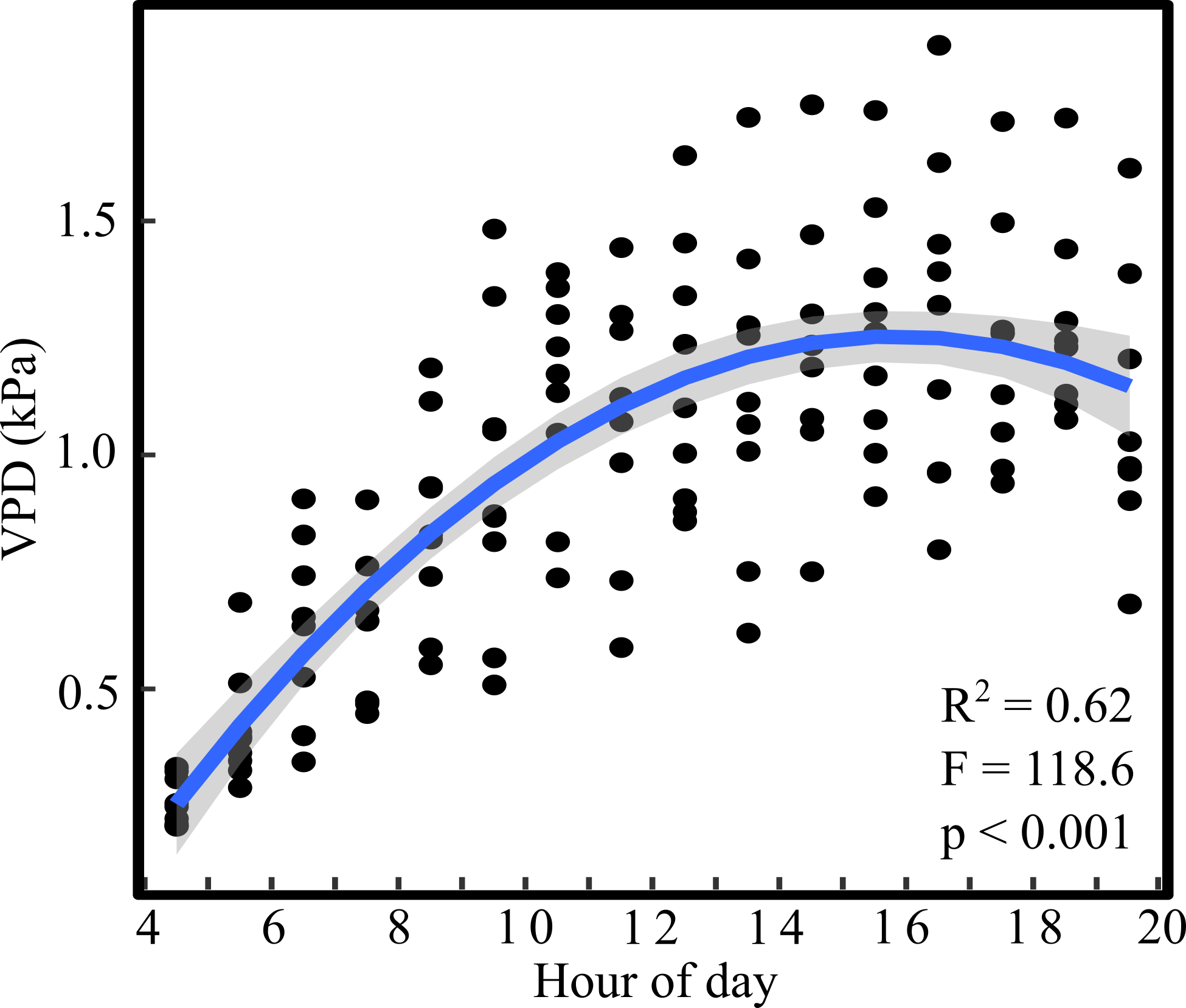
**

**Fig. S5** Water-use efficiency estimated from ∆^13^C (*WUE*_∆_) by the simple model.

*WUE*_∆_ was compared to water-use efficiency calculated from continuous gas-exchange data (*WUE*_G_). Red dots are data points between 04:00 and 08:00, and black dots are data points between 08:00 and 20:00. The points represent cuvette means (*n* = 4) for each hour and day. The blue line represents the regression fit to the black dots, and the shaded grey area is the standard error of the fit. The red line represents the theoretical 1:1 fit for comparison.

**
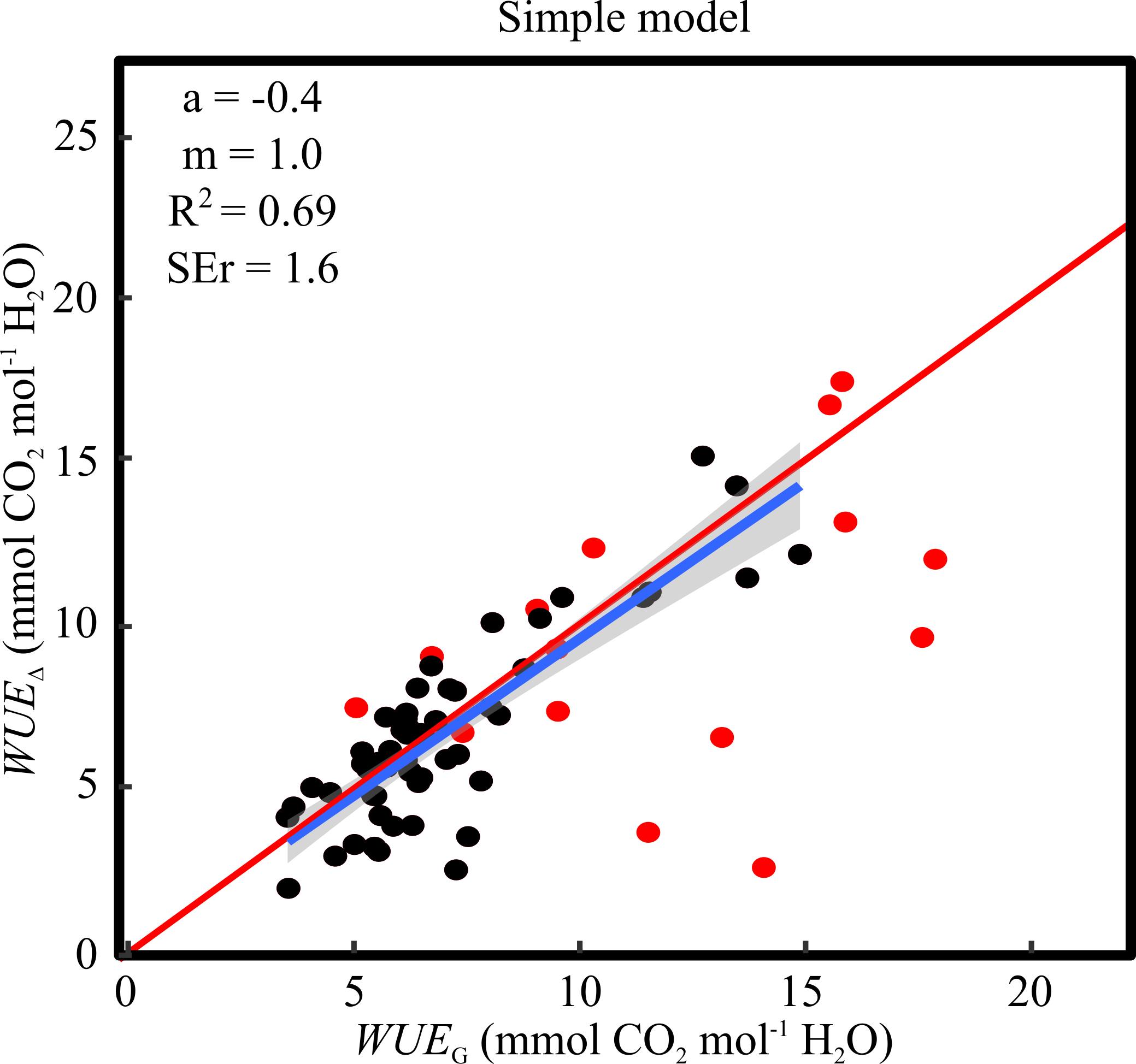
**

**Fig. S6** Water-use efficiency estimated from ∆^13^C data (*WUE*_∆_) by the comprehensive model; accounting for *g*_m_ as a) constant *g*_m_/*g*_s_ and b) infinite *g*_m_.

*WUE*_∆_ was compared to water-use efficiency calculated from continuous gas-exchange data (*WUE*_G_). The points represent the cuvette means (*n* = 4) for each hour and day. The blue line represents the regression fit to the data, the shaded grey area is the standard error of the fit. The red line represents the theoretical 1:1 fit for comparison.

**
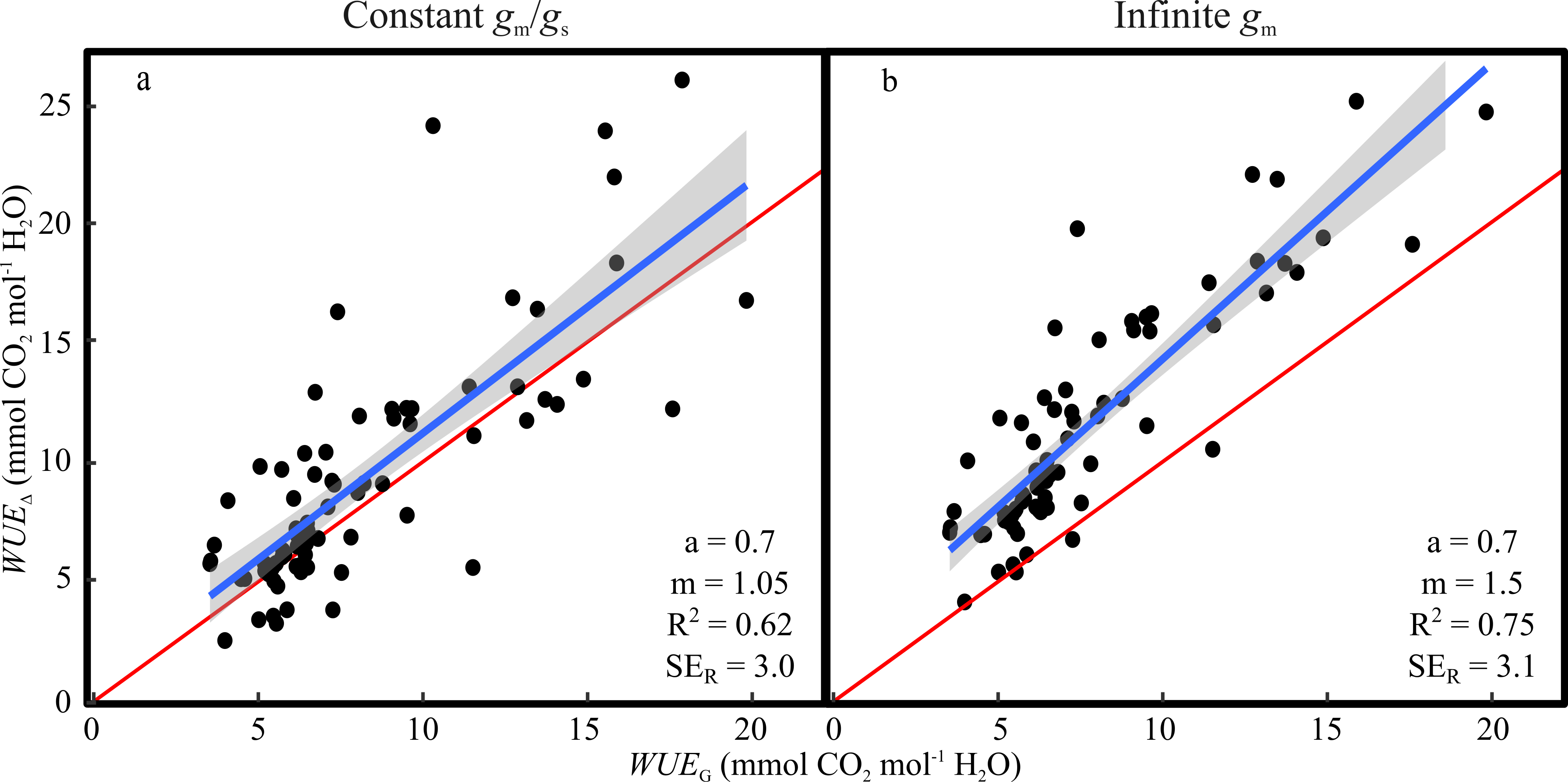
**

**Fig. S7** Diurnal variation of δ^13^C of *A*_net_ (Eqn S4)

The points represent the cuvette means (*n* = 4) for each hour and day during the measurement period. The blue line is the second order polynomial fit to the data and the shaded grey area is the standard error of the fit.


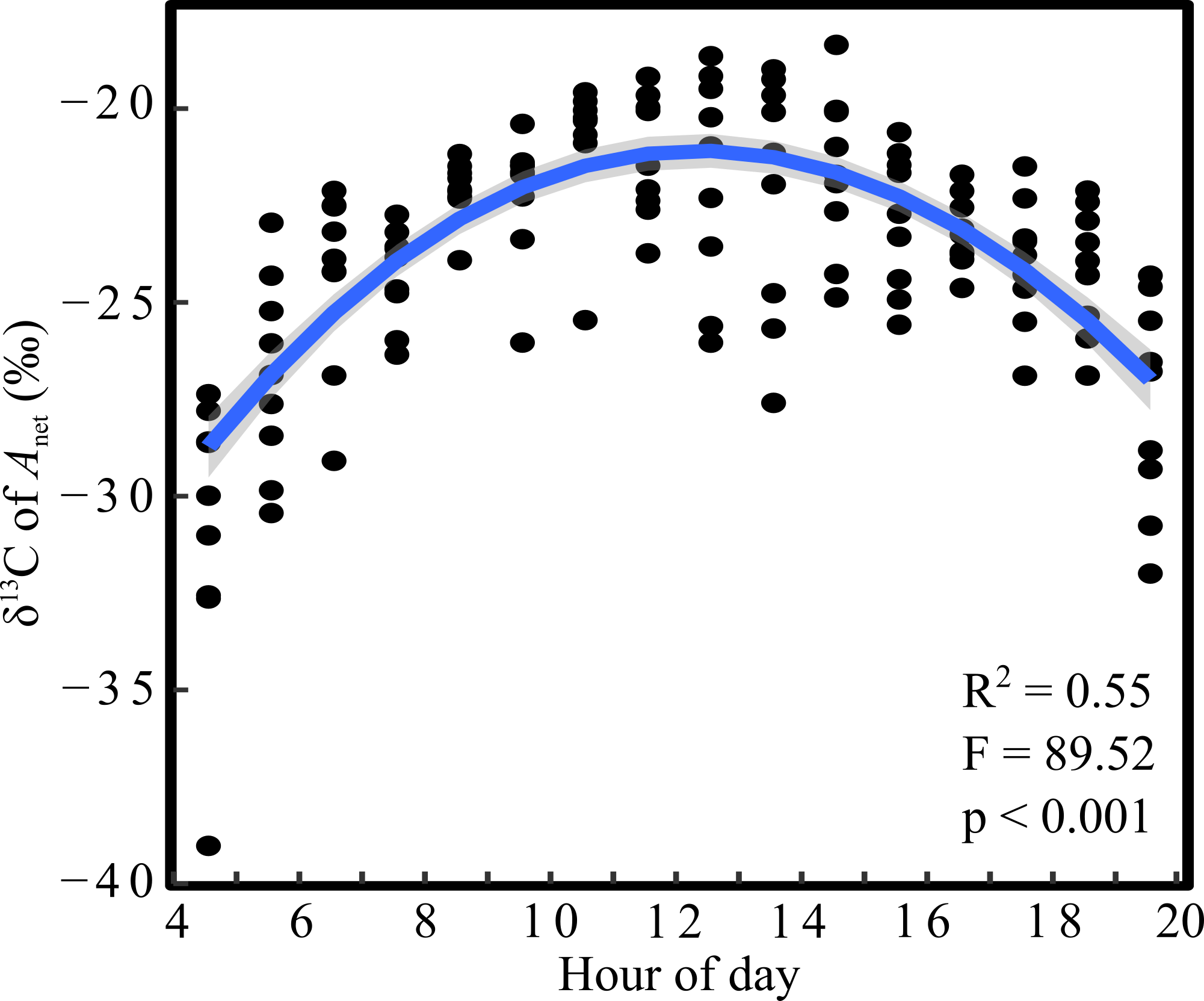


**Fig. S8** Diurnal variation in *WUE* estimated from continuous gas-exchange data

The points represent the cuvette means (*n* = 4) for each hour and day during the measurement period. The blue line is the second order polynomial fit to the data and the shaded grey area is the standard error of the fit.


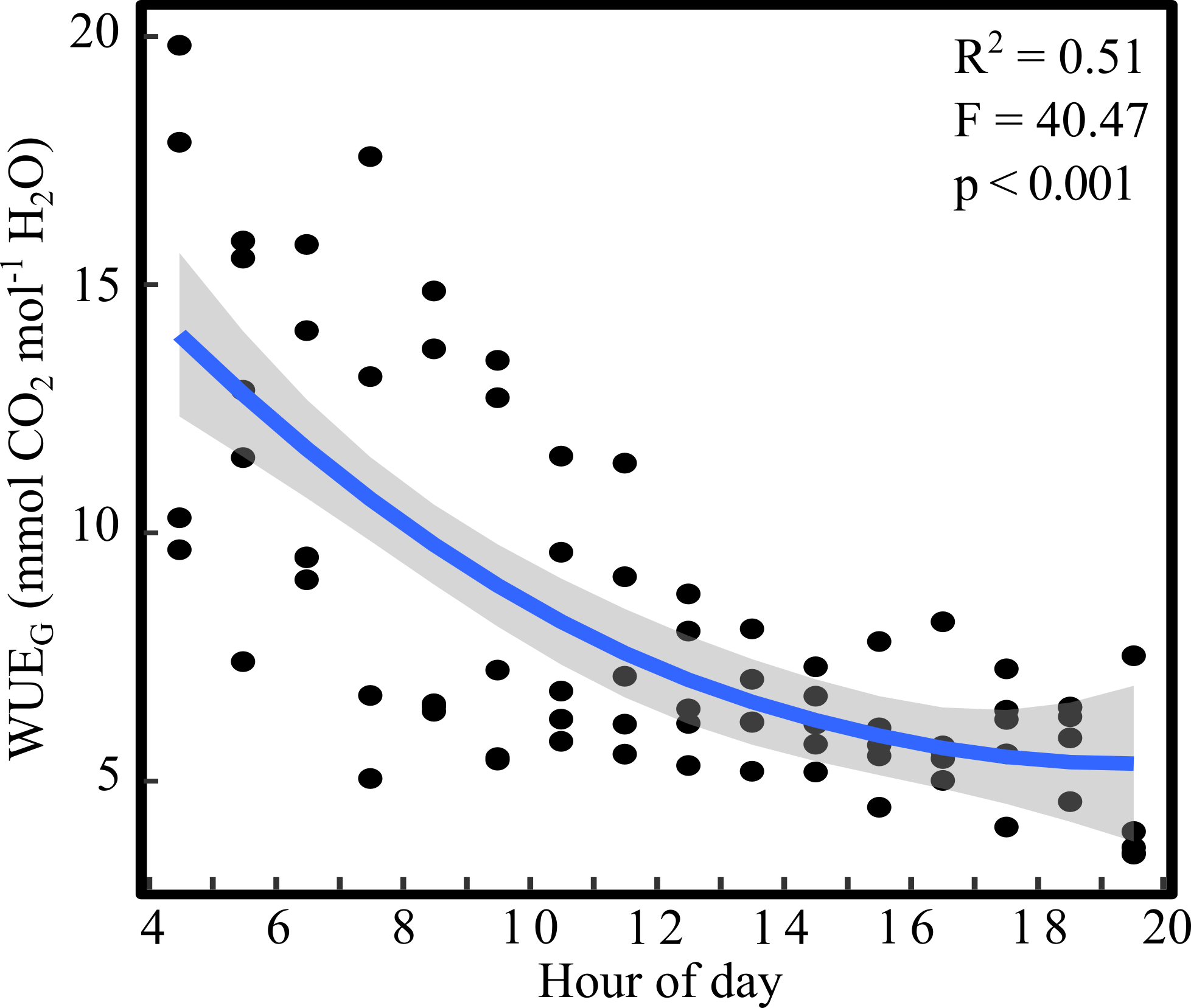


**Supplementary Materials and Methods**

*Daytime respiration*

Reference daytime respiration (*R*_dRef_ = 0.271 µmol m^-2^ s^-1^) was assumed to be lower than at night and it was calculated as *R*_dRef_ = *R*_night_ * 0.6, where *R*_night_ is the mean rate of respiration (0.452 ± 0.06, mean ± standard error) during the darkest hour of the night (23:00 - 00:00). The temperature response of *R*_d_ was estimated with a polynomial response curve proposed by Heskel et al. for evergreen conifers (Heskel et al. 2016), as follows:

$$R_{d}=R_{\mathrm{dRef}}\times e^{\left[ 0.0864\left( T - T_{\mathrm{Ref}} \right)-0.00013\left( T^{2}-T_{\mathrm{Ref}}^{2} \right) \right]}$$

(S1)

where *T*_Ref_ = 9.4 °C is the reference temperature for *R*_dRef_ and T is the actual temperature in the leaf cuvette.

*^13^C discrimination during photosynthesis*

The component discriminations in Eqn.1 were calculated according to Evans & von Caemmerer (2013) and Farquhar & Cernusak (2012).

Discrimination when *C*_i_ = *C*_c_:

$$\begin{matrix} \text{Δ}_{i}= & \frac{1}{1-t}a_{s}+\frac{1}{1-t}((1+t)b-a_{s})\frac{C_{i}}{C_{\mathrm{out}}} \end{matrix}$$

(S2)

where *a*_s_ is the fractionation of the diffusion through air (4.4‰), *b* fractionation during carboxylation (*b* = 29‰), *C*_out_ and *C*_i_ are the CO_2_ concentrations inside the cuvette and the leaf intercellular spaces, respectively, and *t* is the ternary correction (Eqn S7; Farquhar and Cernusak, 2012).

Observed discrimination:

$$\Delta_{o}=\frac{\delta^{13}C_{\mathrm{out}}-\delta^{13}C_{\mathrm{Anet}}}{1+\frac{\delta^{13}C_{\mathrm{Anet}}}{1000}}$$

(S3)

where δ^13^*C*_out_ and δ^13^*C*_Anet_ are the δ^13^C of the air flow out of the cuvette and photosynthesis respectively and,

$$\delta^{13}C_{\mathrm{Anet}}=\frac{(\delta^{13}C_{\mathrm{in}}\times C_{\mathrm{in}})-(\delta^{13}C_{\mathrm{out}}\times C_{\mathrm{out}})}{C_{\mathrm{in}}-C_{\mathrm{out}}}$$

(S4)

where δ^13^C_in_ is the δ^13^C of the air flow into the cuvette and *C*_in_ and *C*_out_ are the CO_2_ concentrations of the air flow into and out of the cuvette, respectively. In Eqn S4 the CO_2_ concentrations (*C*_in_ and *C*_out_) are corrected for water vapour concentration and are expressed as µmol mol^-1^ dry air. Note that the rapid stirring inside the cuvette means that C_out_ and δ^13^C_out_ represent the “ambient” conditions within the cuvette. These conditions are not, however, the same as ambient conditions outside the cuvette because the turnover of cuvette air is rather slow and photosynthesis has time to modify its composition.

Discrimination associated with respiration:

$$\begin{matrix} \Delta_{e}= & \frac{1+t}{1-t}\frac{eR_{d}}{A_{\mathrm{net}}+R_{d}}\frac{C_{i}-\Gamma^{*}}{C_{\mathrm{cuv}}} \end{matrix}$$

(S5)

where Γ*** is the CO_2_ compensation point, derived from an Arrhenius function (Bernacchi et al. 2001; Medlyn et al. 2002 Eqn. 12), and the respiratory fractionation *e* is calculated as:

$$e=e_{\mathrm{Rd}}+e^{*}=-2 + (\delta^{13}C_{a}-\Delta_{o}-\delta^{13}C_{su\mathrm{bstrate}})$$

(S6)

where we assumed that δ^13^*C*_substrate_ = δ^13^*C*_Anet_ (Wingate et al. 2007, Gessler et al. 2008, Farquhar and Cernusak 2012).

Discrimination associated with photorespiration:

$$\begin{matrix} \Delta_{f}= & \frac{1+t}{1-t}f\frac{\Gamma^{*}}{C_{\mathrm{out}}} \end{matrix}$$

(S7)

where *f* = 16.2 ‰ (Evans and von Caemmerer 2013) is the fractionation factor of photorespiration.

The ternary correction factor (*t*) was calculated assuming infinite boundary layer conductance as:

$$t=\frac{(1+a_{a})E}{2g_{s}}$$

(S8)

where *E* is the rate of transpiration (mol H_2_O m^-2^ s^-1^).

*Models for Δ^13^C*

The comprehensive Δ^13^C model, written here assuming infinite boundary layer conductance and ignoring α-terms, is (Farquhar et al. 1982, Farquhar and Cernusak 2012):

$$\Delta^{13}\text{C}_{\text{com}}=\frac{1}{1-t}\left[ a_{s}\frac{C_{\text{out}}-C_{\text{i}}}{C_{\text{out}}} \right]+\frac{1+t}{1-t}\left[ a_{\text{m}}\frac{C_{\text{i}}-C_{\text{c}}}{C_{\text{a}}}+b\frac{C_{c}}{C_{out}}-e\frac{R_{d}}{A_{net}+R_{d}}\frac{C_{c}-\Gamma^{*}}{C_{out}}-f\frac{\Gamma^{*}}{C_{\text{out}}} \right]$$

(S9)

Solving *C*_i_ from Eqn S9 results in the quadratic equation (Cernusak et al., 2018):

$$C_{i}= \frac{-II \pm\sqrt{{II}^{2}-4I*III}}{2I}$$

for which the solution is written here for the case when boundary layer conductance is assumed infinite:

$$I = a_{s}(\frac{eR_{d}}{A_{net}+R_{d}}-b-1)$$

(S10)

$$II = \Delta_{o}C_{out}(-2-a_{s})-a_{s}\left[ \frac{A_{net}}{g_{m}P}(a_{m}-b+\frac{eR_{d}}{A_{net}+R_{d}})+(\frac{eR_{d}}{A_{net}+R_{d}}-f)\Gamma^{*}+\frac{2A_{net}}{E} \right]+\left[ 2C_{out}+\frac{2A_{net}}{E}+a_{s}C_{out} \right](b-\frac{eR_{d}}{A_{net}+R_{d}})$$

(S11)

$$III = a_{s}C_{out}(C_{out}+\frac{2A_{net}}{E})-\Delta_{o}C_{out}(\frac{2A_{net}}{E}-a_{s}C_{out})+\left[ C_{out}(2+a_{s})+\frac{2A_{net}}{E} \right]\left[ \frac{A_{net}}{g_{m}P}(a_{m}-b+\frac{eR_{d}}{A_{net}+R_{d}})+(\frac{eR_{d}}{A_{net}+R_{d}}-f)\Gamma^{*} \right]$$

(S12)

Assuming that the contribution of respiratory fractionation to the total discrimination is negligible and ignoring ternary corrections, Eqn S9 simplifies to what we termed the partial model, which is:

$$\Delta^{13}\text{C}_{\text{par}}=a_{s}\frac{C_{out}-C_{i}}{C_{out}}+a_{m}\frac{C_{i}-C_{c}}{C_{out}}+b\frac{C_{c}}{C_{out}}-f\frac{\Gamma^{*}}{C_{out}}$$

(S14)

In Eqn S14, *C*_c_ can be substituted by $C_{i}-\frac{A}{g_{m}}$ and subsequently the term $\frac{A}{g_{m}C_{out}}$ can be replaced with $\frac{g_{sw}}{1.6g_{m}}\left( 1-\frac{C_{i}}{C_{out}} \right),$ where *g*_sw_ is the stomatal conductance to water. From the resulting expression the *C*_i_ can be solved, which results in Eq 5 in the main text (Seibt, 2008).

Finally, assuming *g*_m_ infinite and ignoring the contribution of photorespiration, Eqn S14 simplifies to the simple model, which is:

$$\Delta^{13}\text{C}_{\text{sim}}=a_{s}+\left( b-a_{s} \right)\frac{C_{i}}{C_{a}}$$

(S15)

The *C*_i_ can be solved from Eqn S15, which results in Eqn 6 in the main text.
